# An Integrated mHealth App for Dengue Reporting and Mapping, Health Communication, and Behavior Modification: Development and Assessment of Mozzify

**DOI:** 10.2196/16424

**Published:** 2020-01-08

**Authors:** Von Ralph Dane Marquez Herbuela, Tomonori Karita, Micanaldo Ernesto Francisco, Kozo Watanabe

**Affiliations:** 1 Graduate School of Science and Engineering Ehime University Bunkyo-cho 3 Matsuyama Japan; 2 Department of Special Needs Education, Faculty of Education Ehime University Bunkyo-cho 3 Matsuyama Japan; 3 Biological Control Research Unit, Center for Natural Science and Environmental Research De La Salle University Manila Philippines

**Keywords:** dengue fever, mHealth, real-time surveillance, health communication, behavior modification

## Abstract

**Background:**

For the last 10 years, mobile phones have provided the global health community with innovative and cost-effective strategies to address the challenges in the prevention and management of dengue fever.

**Objective:**

The aim is to introduce and describe the design and development process of Mozzify, an integrated mobile health (mHealth) app that features real-time dengue fever case reporting and mapping system, health communication (real-time worldwide news and chat forum/timeline, within-app educational videos, links to local and international health agency websites, interactive signs and symptoms checker, and a hospital directions system), and behavior modification (reminders alert program on the preventive practices against dengue fever). We also aim to assess Mozzify in terms of engagement and information-sharing abilities, functionality, aesthetics, subjective quality, and perceived impact.

**Methods:**

The main goals of the Mozzify app were to increase awareness, improve knowledge, and change attitudes about dengue fever, health care-seeking behavior, and intention-to-change behavior on preventive practices for dengue fever among users. It was assessed using the Mobile Application Rating Scale (MARS) among 50 purposively sampled individuals: public health experts (n=5), environment and health-related researchers (n=23), and nonclinical (end users) participants (n=22).

**Results:**

High acceptability and excellent satisfaction ratings (mean scores ≥4.0 out of 5) based on the MARS subscales indicate that the app has excellent user design, functionality, usability, engagement, and information among public health experts, environment and health-related researchers, and end users. The app’s subjective quality (recommending the app to other people and the app’s overall star rating), and specific quality (increase awareness, improve knowledge, and change attitudes about dengue fever; health care-seeking behavior; and intention-to-change behavior on preventive practices for dengue fever) also obtained excellent satisfaction ratings from the participants. Some issues and suggestions were raised during the focus group and individual discussions regarding the availability of the app for Android devices, language options limitations, provision of predictive surveillance, and inclusion of other mosquito-borne diseases.

**Conclusions:**

Mozzify may be a promising integrated strategic health intervention system for dengue fever case reporting and mapping; increase awareness, improve knowledge, and change attitude about dengue fever; and disseminating and sharing information on dengue fever among the general population and health experts. It also can be an effective aid in the successful translation of knowledge on preventive measures against dengue fever to practice.

## Introduction

For the last 10 years, mobile phones have provided the global health community with innovative and cost-effective strategies to address the challenges in the prevention and management of dengue fever [1]. Dengue fever, which is considered an international public health concern, especially in tropical and subtropical countries, puts an estimated 2 to 3.97 billion people at risk of hospitalization and even death [2,3].

Mobile health (mHealth) is a concept that uses mobile communication devices, such as mobile phones, to deliver services through mobile apps [1]. Apps are specialized software programs that are often equipped with the capability to link to internet sources and services, including health care providers [1]. App-enabled mHealth is emerging as the driver for next-generation telemedicine and telehealth [1]. However, there is a lack of apps that address the prevention and control of dengue fever with relevant studies.

To our knowledge, only one app has been developed with relevant studies, Mo-Buzz. It is a mobile pandemic surveillance system for dengue fever with three main components: predictive surveillance, civic engagement, and health communication [4,5]. These components address the three main limitations in the control and management of dengue fever: (1) use of traditional epidemiological methods (eg, failure to identify the “turning point” of the outbreak leads to vector control measures such as carpet-combing [“search-and-destroy” mosquito breeding sites] near or at the peak of transmission be less impactful [6]), which leads to reactive or poor disease monitoring and surveillance; (2) lack of participation from the public; and (3) lack of effective and interactive health education for the public, which prevents successful translation of awareness or knowledge into actions [4].

Although Mo-Buzz was found to play a significant role in the management and control of dengue fever, we have developed a different mobile app, Mozzify, which offers an integrated mHealth system to address the challenges in dengue fever prevention and management. It has three components: real-time surveillance, health communication, and behavior modification. The main component is the real-time surveillance feature for reporting and mapping dengue fever cases (both laboratory-confirmed hospital and probable dengue fever cases) and mosquito bites. Compared with Mo-Buzz, Mozzify reports and maps dengue fever cases and mosquito bites in real time (versus predictive surveillance) through an online Web map system. There is a lack of spatiotemporal data for dengue fever cases; the data from the real-time surveillance will serve as springboard data for combined predictive and real-time reporting and mapping features of the app in the future. These will be helpful to identify dengue fever hotspots (locations with high incidences of dengue fever cases), so health officials can deliver prompt and early warning communication as well as awareness to the public who are at risk of contracting the disease. Another difference is that Mozzify not only allows reports of probable cases of dengue fever and mosquito bites but also allows reporting of laboratory-confirmed dengue fever cases. Kao et al [7] recommended the introduction of a holistic surveillance system (eg, clinical, serological, and virological) to prevent large-scale epidemics and severe dengue fever cases. The study also recommended the use of a geographical information system for spatial analysis and epidemic prediction models [7].

Another difference of Mozzify from Mo-Buzz is the inclusion of some features in the health communication component. We have developed a system that reports real-time worldwide news about dengue fever and other mosquito-borne diseases; within-app educational videos on the diagnosis, treatment, and management of dengue fever and control of vector mosquitoes; links to websites of local and international health agencies; and a real-time timeline chat forum for sharing information among users. These features aim to increase the public’s awareness of the signs and symptoms, treatment, and management of dengue fever as well as the prevention and control of vector mosquitoes.

In addition to these features that center on real-time data, two other unique features of Mozzify that differentiate it from Mo-Buzz are the signs and symptoms checker and the interactive hospital directions. We designed a system that lets users check their signs and symptoms of dengue fever and identify the hospitals that have dengue fever express lanes and cater to Dengvaxia-vaccinated individuals. The aim is to not only inform users about the signs and symptoms of dengue fever but also motivate their health care-seeking behavior, which is based on the health belief model.

The health belief model is a widely used social cognition model to predict health behaviors. This model suggests that a change in behavior or action can be expected if a person perceives themselves to be at risk or susceptible to the disease (perceived susceptibility), that the disease will have serious consequences (perceived severity), a course of action will minimize consequences (perceived benefits), and the benefits of action will outweigh the cost of barriers (perceived barriers) and self-efficacy [8]. However, barriers to sustained self-prevention against dengue fever are caused by a lack of self-efficacy, lack of perceived benefit, and low perceived or unsure susceptibility [9]. People who perceive themselves at risk of dengue fever visit a health care provider promptly compared with those who perceive the opposite [10]. Health care-seeking behavior is also greatly influenced by the inadequacy of primary health care facilities in giving adequate services to dengue fever patients [11].

More importantly, what makes Mozzify different from Mo-Buzz is the inclusion of behavior modification as an important component to address the poor translation of awareness or knowledge of the different preventive practices against dengue fever into actions. To address this, we added a feature that allows users to choose and add items on the list of preventive practices against dengue fever and directly transmit it to the built-in iPhone iOS Reminders app to set-up dates and locations of alerts. It has been reported that epidemiology is strongly associated with human habits and activities [12]. Kumaran et al [13] and Shuaib et al [14] reported that knowledge of the causes, signs, symptoms, mode of transmission, and preventive practices against dengue fever is not correlated with the practice of preventive measures against dengue fever. Thus, health programs should be designed to focus on translating knowledge into better and effective practices against dengue fever through behavior change. Many programs continue to focus only on changing people’s knowledge or raising awareness rather than physical activity programs, which are more successful at producing behavior change [15]. We have used the concept behind Communication for Behavioral Impact (COMBI), a comprehensive strategy that uses communication for knowledge to have a significant effect on behavioral change (making people aware, informed, convinced, and decide to act, then repeating and maintaining that action) to increase the practice of preventive measures against dengue fever [13,16].

This paper aims to describe the design and development process of the Mozzify app*.* We will also assess it in terms of engagement and information-sharing abilities, functionality, aesthetics, subjective quality, and perceived impact among public health experts, environment and health-related researchers, and nonclinical or general public participants (end users). We hypothesize that the participation and acceptance rates (user’s intention to use the app) among the participants will be high due to the app’s relevance to the dengue fever control and health communication program. We also hypothesize that the majority of participants will perceive that users will have increased awareness, improved knowledge, and changed attitudes about dengue fever, which will increase health care-seeking behavior and behavior change (on preventive practices against dengue fever) through the use of the app.

## Methods

### Mozzify App Design and Development

Mozzify is an integrated mHealth app for dengue fever cases reporting and mapping, health communication, and behavior modification. It was developed for the iOS mobile phone platform using Xcode (versions 10.1 to 11.0) software in Swift (versions 4.2 to 5) programming language. The name, Mozzify, is based on the word *mosquito* because its primary purpose is to collect and disseminate information about dengue fever, which is a viral infection transmitted by mosquitoes.

**Figure 1:**
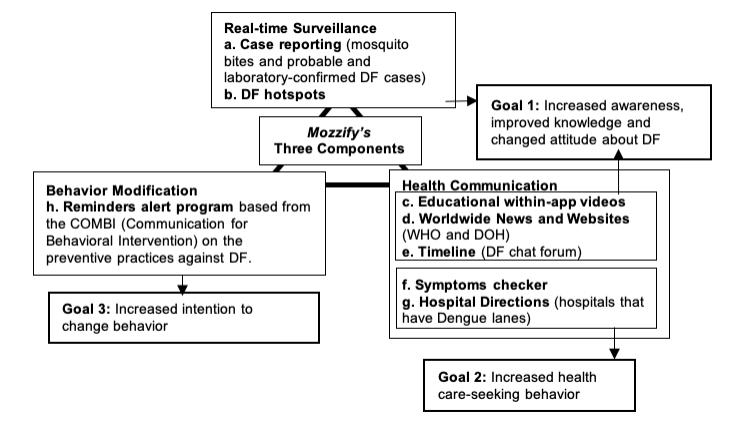
Mozzify’s three components with their corresponding features and goals. DF: dengue fever; DOH: Department of Health; WHO: World Health Organization.

**Figure 2:**
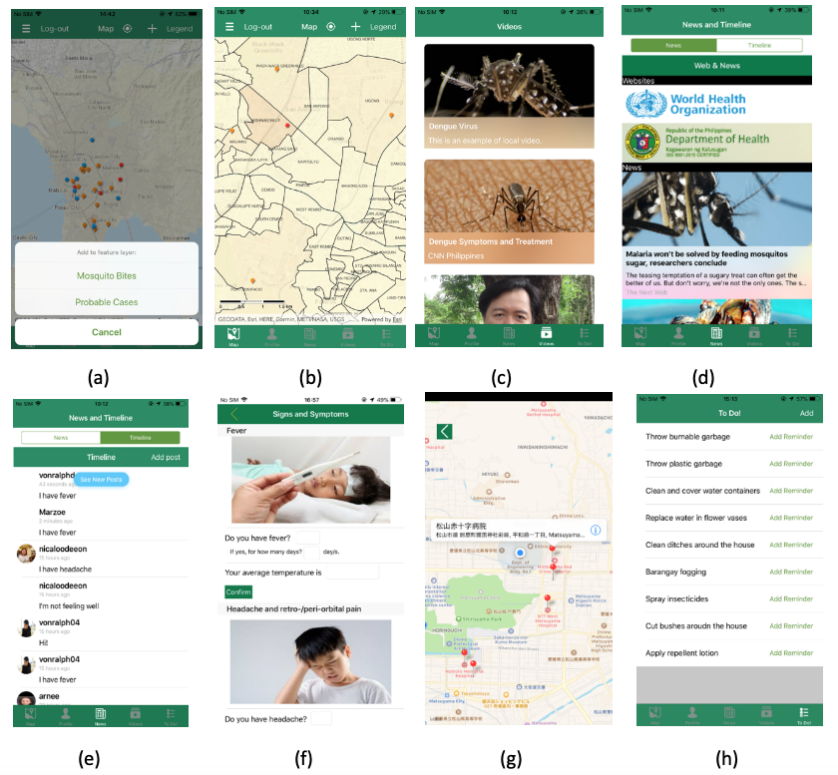
Screenshots of the Mozzify app. (a) Real-time dengue fever cases and mosquito bite reporting and mapping, (b) dengue fever hotspots, (c) within-app educational videos, (d) worldwide news and health agencies websites, (e) chat forum (timeline), (f) symptoms checker, (g) hospital directions, and (h) reminders alert program.

Mozzify has three components: (1) real-time dengue fever cases reporting and mapping, (2) health communication, and (3) behavior modification. These components were matched to three main goals: (1) increase awareness, improve knowledge, and change attitude about dengue fever; (2) increase health care-seeking behavior; and (3) increase intention-to-change behavior on preventive practices against dengue fever. Figure 1 shows the app’s three components and goals with its corresponding features. Screenshots of some of the features are shown in Figure 2.

### Sign-Up

Sign-up required users to provide information (eg, username and photo [optional], email, and password) to access the app’s features. The app collects, stores, and uses personally identifiable information through Firebase, a third-party service provider that serves as our database. The app will collect, store, and use some identifiable information from users to provide its services; therefore, we generated our own privacy policy and terms and conditions (see the Mozzify app user guide Multimedia Appendix 1). Agreement to these was necessary to proceed with sign-up.

### Component 1: Real-Time Dengue Fever Cases Reporting and Mapping System

The main feature of this app is the real-time reporting and mapping of dengue fever cases and mosquito bites through ArcGIS Online. ArcGIS Online is an online, cloud-based, collaborative and configurable Web geographical and information system that allows developers to use, create, analyze, and share maps in mobile apps [17]. To use the ArcGIS Online features in our app, we configured the source code provided by Data Collection.NET, which required the installation of ArcGIS Runtime SDK (version 100.5) [18]. Configuration involved registration of our own ArcGIS Portal app, modifying the project in reference to our app, and licensing it for deployment [19]. The map shows real-time probable and laboratory-confirmed dengue fever cases and mosquito bite reports in pins. Probable cases are in blue pins, confirmed dengue fever cases are in red pins, and mosquito bite reports are in orange pins. Users will be able to report a probable case or a mosquito bite incidence, which they can pin at their current location (or any other location). In reporting, users can disclose their personal information (eg, name, home address, and age) and attach images. The map also shows the location (*barangay* or village) with high dengue fever incidences by color using the ArcGIS analysis feature. This feature assigns a darker color to a location with high dengue fever incidences by counting the number of pins (confirmed and probable dengue fever cases) within its boundary. This analysis is done on a daily basis.

### Component 2: Health Communication

The unique parts of the health communication component are the dengue fever warning signs and symptoms checker and the hospital directions feature. Users can answer 26 simple questions (three questions per symptom) in each symptom (eg, Do you have fever? If yes, for how many days? What is your average temperature?), and the app alerts the user if the symptoms need prompt clinical attention by a physician. We formulated the questions and set an algorithm based on the clinical diagnosis, treatment, and management guidelines of dengue fever to allow the app to analyze whether the user needs to go to the nearest hospital to receive prompt clinical assessment by a health professional based on their answers (eg, a fever above 41°C for four days requires immediate clinical assessment by a physician) [2]. If the user’s symptoms require prompt medical assessment by a physician, the app sends an alert that the user needs to go to the hospital. Then the app will present a map that shows the user’s current location and the nearest hospitals. We collected the coordinates (latitude and longitude) of each hospital and pinned them as an annotation in the map using Google Maps. Once the user clicks a pin of a hospital, the app then shows the directions from their current location to the chosen hospital. In this trial, we selected random nearby hospitals to show the app’s feature. The app is intended to be trialed in the Philippines; therefore, we used the list of the hospitals from the Department of Health that has dengue express lanes (including for Dengvaxia-vaccinated individuals who might need immediate medical assistance and free hospital services) [20]. This feature aims to increase health care-seeking behavior among users by encouraging them to go to the hospital to seek medical help from health care professionals and not to self-diagnose or, more importantly, cause a panic.

The app contains different online-sourced, evidence-based, local and international guidelines on the control, prevention, diagnosis, and treatment of dengue fever in PDF (portable document format) files [2,21-24]. Users can also watch predownloaded videos in English and Filipino (Tagalog) on the dengue fever virus, symptoms, diagnosis, and treatment available. We also used news Application Programming Interface (API), a free, open-source, and noncommercial API that collects news from our set request parameters (eg, keywords: mosquito, dengue fever) and shows users the latest international and local news from almost 30,000 news sources and blogs on dengue fever and other mosquito-borne diseases, such as malaria, zika, chikungunya, and Japanese encephalitis. The app also shows the websites of international and local health agencies, such as the World Health Organization and Department of Health, which give the users information on key facts, prevalence, treatment, immunization, prevention, and control guidelines and programs on dengue fever [25,26]. The app’s health communication feature was also designed to let users exchange and share posts on events, concerns, and questions on dengue fever through a chat forum (timeline) by using the Firebase online database. Altogether, these aim to increase awareness, improve knowledge, and change attitudes about dengue fever.

### Component 3: Behavior Modification (Preventive Practices Against Dengue Fever)

Another component of the app is the behavior modification feature, which includes the Reminder alerts program based on COMBI. This is expected to develop or improve users’ behavior on practicing preventive measures against dengue fever. COMBI is a comprehensive strategy that uses communication for knowledge to have a significant effect on behavioral change (making people aware, informed, convinced, and then deciding to act, and repeating and maintaining that action) or increase practices against dengue fever [13,16]. The app has a list of practices against dengue fever based on international and local guidelines on prevention, management, and vector control programs [2,21-24]. The user has the option to select and add practices according to their needs or preference. After selection, the app adds all the items to the built-in iOS Reminders app on their mobile phones. In that app, users can set the priority and edit when (eg, time, day, and frequency [daily, weekly, or monthly]) and where (radial location settings) they want alerts.

### Testing and Assessment

This study was written and conducted in accordance with international guidelines: the Declaration of Helsinki [27] and the International Council for Harmonization Good Clinical Practice guidelines [28]. The protocol was approved by the Ethics and Review Committee of Ehime University, Japan (ethics review approval number: K19-001). The app was tested in July 2019 after the completion of its development.

This study involved 50 purposively sampled participants grouped into three subgroups who tested the app: (1) public health experts (n=5), (2) environment and health-related researchers (n=23), and (3) nonclinical users (n=22). The participants were selected, recruited, and grouped according to the set inclusion criteria. Public health experts were academic and clinical research experts working directly on prevention, control (including an ArcGIS mapping expert), and clinical management of dengue fever. Environment and health-related researchers were university-based researchers working on vector-borne diseases and water ecology. Both the public health experts and environment and health-related researchers were from countries where dengue fever is prevalent (eg, the Philippines and Indonesia). The third subgroup, nonclinical, were considered as end users and had no comprehensive knowledge of or experience with dengue fever. A few information technology and computer science researchers were also included in the environment and health-related researchers and nonclinical groups.

All participants were aged 18 years and older, with sound psychological conditions, who were able to read and understand the informed consent contents, and used an iPhone mobile phone of any model with iOS of 11.0 and above. Initially, when found eligible, participants were asked to sign an informed consent sheet. Of the 50 participants, 36 (72%) were invited for either a focus group or an individual discussion session to provide more comprehensive qualitative feedback, whereas others, who were mostly in the nonclinical group, were invited by email (n=14). All participants were asked to install the app on their mobile phones after a Web-based invitation by downloading Test Flight, the beta testing feature of the Apple Inc developer program. After installation, all participants were asked to use the app by following the instructions in the user’s guide (see Multimedia Appendix 1), which was available in print and on a website.

Participants were asked to complete the Mobile Application Rating Scale (MARS). Their responses were automatically stored in the password-protected database, and each participant was given a unique code to protect their identity. MARS is a 23-item test that uses a 5-point scale (1=inadequate, 2=poor, 3=acceptable, 4=good, 5=excellent), which assesses the app quality on three subscales: objective quality, subjective quality, and app-specific quality [29]. The objective quality subscale has 19 items that are clustered into four parts: engagement (fun, interesting, customizability, interactivity, and well-targeted to audience), functionality (functioning, easy-to-learn, navigation, flow logic, and gestural design), aesthetics (graphic design, overall visual appeal, color scheme, and stylistic consistency), and information quality (containing high-quality information from a credible source) [29]. The subjective quality subscale has four items that measure the user’s desire to recommend the app to others, use the app for short or long term, and overall star rating of the app [29]. The app-specific subscale was modified based on the perceived impact of the app on the user’s awareness and knowledge (dengue fever symptoms, hospitals that cater to dengue fever patients, dengue fever hotspots, dengue fever prevention and treatment), attitudes (perceived risk or susceptibility, perceived severity, benefits and barriers, and self-efficacy), help-seeking behavior (health care-seeking behavior), intentions, and actual change of behavior in practicing preventive measures against dengue fever. Scoring was done by calculating the mean score of each subscale, adding them together, and dividing by four (four subscales) to get the app quality mean score, four (four items) to get the app subjective quality mean score, or six (six items) to get the app-specific mean score [29]. Irrelevant items were one item in the information subscale (if the app underwent evidence-based trial/test) and one item in the subjective quality subscale (Would you pay for this app?) and these were not included in the analysis (app has not been trialed/tested and will not be sold). MARS has an excellent total score internal consistency level Cronbach alpha (α=.90) and excellent level of interrater reliability (two-way mixed interclass correlation coefficient [ICC]=0.79, 95% confidence interval [CI] 0.75-0.83). Its subscales also had very high internal consistencies that ranged from alphas of .80 to .89, and fair to excellent interrater reliabilities (ICC 0.50-0.80, median 0.65) [29]. The MARS form is available in Multimedia Appendix 2.

### Data Analysis

Statistical analysis was conducted using SPSS version 25 (IBM Corp, Armonk, NY, USA). We calculated the mean scores of each subscale in the app objective quality scale and each item in the app subjective scale. The app subjective quality scale was reported as individual items and by mean score. Comments and suggestions in the focus group and individual discussions were analyzed, and similar or overlapping topics were grouped into different themes.

## Results

### Participants

A total of 50 individuals, aged from 19 to 45 years and mostly males (60%, 30/50), participated in the app testing. All participants we approached and recruited agreed to test the app and answer the scale; the majority (78%, 39/50) attended the focus group and individual discussions. This indicates excellent acceptability and nonwithdrawal rates among experts, health researchers, and the general public or end users. In all, 68% (34/50) were able to install the app on their mobile phones, whereas the rest used our available prototypes.

### App Objective Quality

**Figure 3:**
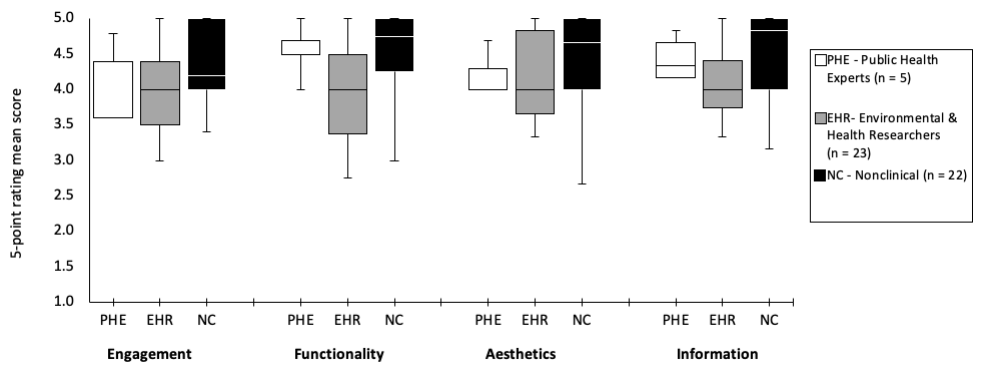
Mean scores of app objective subscales based on the Mobile Application Rating Scale (MARS) from public health experts, environment and health-related researchers, and nonclinical participants.

Mozzify was assessed using the MARS app objective quality, which has four subscales: engagement, functionality, aesthetics, and information. Figure 3 shows the mean score ratings of the public health experts, environment and health-related researchers, and nonclinical or general public participants. Mozzify received high (≥4 out of 5; 74%, 37/50) satisfaction mean ratings in all four subscales among the participants, with the highest mean score ratings (mean 4.3, SD 0.2) for items relating to the app’s functioning, easiness to learn, navigation, flow, logic, and gestural design*,* graphic design, overall visual appeal, color scheme, and stylistic consistency*,* and for containing high-quality information from a credible source. A mean rating of 4.2 (SD 0.2) was obtained for items relating to the app being fun, interesting, customizable, interactive, and well-targeted to the audience.

### App Subjective Quality

**Figure 4:**
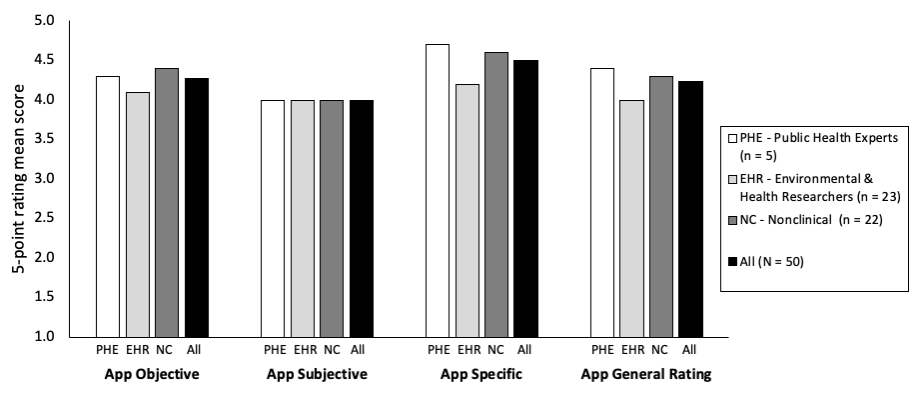
Mean scores of app objective, subjective, specific, and general rating based on the Mobile Application Rating Scale (MARS) from public health experts, environment and health-related researchers, and nonclinical participants.

Public health experts, environment and health-related researchers, and nonclinical participants had similar satisfaction mean ratings (mean 4.0, SD 0.4) in the app subjective quality subscale, as shown in Figure 4. Specifically, items about recommending the app to other people and the app’s overall star rating had relatively high satisfaction mean ratings of 4.2 (SD 0.2), whereas the item on using the app in the next 12 months obtained the lowest satisfaction mean rating of 3.4 (SD 0.3) among the participants. All participants reported that they would recommend the app to people who might benefit from it. All public health experts and a majority of the environment and health-related researchers (86.9%, 20/23) and nonclinical participants (77.3%, 17/22) perceived that the app was relevant to them and they would use it 3 to more than 50 times in the next 12 months. Public health experts gave the app the highest overall star rating of 4.4 (SD 0.5), whereas both environment and health-related researchers and nonclinical participants gave it 4.1 (SD 0.7 and 0.8, respectively).

### App-Specific Quality

The MARS app-specific quality subscale items were modified based on the components and goals of the app, which were to increase awareness, improve knowledge, change attitudes about dengue fever, and increase intention to change and help-seeking behavior among users. The item on increasing users’ knowledge or understanding of dengue fever symptoms, hospitals that cater to dengue fever patients, dengue fever hotspots, and dengue fever prevention and treatment obtained an excellent satisfaction average mean rating of 4.7 (SD 0.3) (Figure 4). Items on increasing user awareness of the importance of addressing dengue fever symptoms, hospitals that cater to dengue fever patients, dengue fever hotspots, dengue fever prevention and treatment, and encouraging users to seek clinical assessment when they have dengue fever symptoms (if required) obtained an average satisfaction mean rating of 4.5 (SD 0.3 and 0.1, respectively) from all participants. Moreover, items on changing users’ attitudes toward improving practices against dengue fever and increasing users’ intentions or motivation to address behavior change (practicing preventive measures against dengue fever) obtained overall mean ratings of 4.4 (SD 0.3) and 4.3 (SD 0.3), respectively, among the participants. In general, the app obtained a high satisfactory mean rating of 4.5 (SD 0.1) in the app-specific quality subscale among the participants.

### Focus Group and Individual Discussions

Approximately 78% (39/50) of participants were able to attend the focus group and individual discussions. Five public health experts were able to participate in the focus group discussion. All found the app useful and were positive about its concept, components, and goals. However, three issues were raised during the session. The first issue was the availability of the app for iOS (iPhone) mobile phones only. Participants mentioned that it would be better if the app was also available for Android so that everyone with a mobile phone could use it. The second issue was the app was only available in English, and it should also be available in other languages. This would allow the app to be distributed in many countries where dengue fever is highly prevalent. Lastly, one expert mentioned about the ability of the app to do predictive surveillance and identify dengue fever hotspots based on past annual dengue fever incidence reports and not only based on the number of pins in a location (barangay or village).

Environment and health-related researchers also had positive feedback about the app. They found the app concise and relevant, interesting, and convenient. Some perceived that the app was effective in reducing the number of dengue fever patients, and it would help increase user’s awareness of dengue fever, especially those who live in tropical and subtropical countries with high numbers of dengue fever cases. Although the majority of the environment and health-related researchers mentioned that the app was easy to use, some suggested that it should include a within-app user’s guide (eg, pop-ups or labels). Two other suggestions were raised about increasing the engagement ability of the app: (1) the app should include games so people will use it daily and (2) it should include other mosquito-borne diseases (eg, zika, chikungunya, Japanese encephalitis, and malaria) in the real-time reporting and mapping feature of the app. Similar to the public health experts, they also suggested that the inclusion of many languages other than English is highly desirable.

The nonclinical group, who were considered the end users, found the app excellent, useful, exciting, interesting, and helpful for countries that suffer from dengue fever outbreaks. They perceived the app was able to improve their knowledge, attitudes, and awareness by seeing the pictures of symptoms, videos, dengue fever hotspots, and nearby hospitals that cater to dengue fever patients. Specific suggestions were mentioned about the technical and functional details of the app that needed polishing and revisions (eg, buttons and gestures).

## Discussion

We have introduced and described the design and development process and the testing of the functions and features of Mozzify. Results show that it obtained excellent acceptability and satisfaction ratings based on the MARS app quality subscales among the participants. This indicates that it has good user design, functionality, usability, engagement, and contains a relevant information system. The app subjective ratings also indicate that the experts and users are more likely to recommend the app to others and more likely to use it frequently in the future. Moreover, based on the specific quality subscale ratings of the participants, the app achieved what it intended to achieve. It could be a highly effective tool in increasing user’s knowledge and awareness of dengue fever symptoms, hospitals that cater to dengue fever patients, dengue fever hotspots, and dengue fever prevention and treatment; changing user’s attitudes about dengue fever and its symptoms; and encouraging users to seek clinical assessment when they have dengue fever symptoms (if required). It also indicates the app can improve users’ practice of measures against dengue fever, and increase users’ intentions or motivation to address behavior change by practicing preventive measures against dengue fever.

Based on the focus group and individual discussions, participants found the app concise, relevant, interesting, convenient, excellent, useful, and exciting. Some perceived that the app is effective in reducing the number of dengue fever patients, and it will help increase user’s awareness of dengue fever, especially those who live in countries with high incidences of dengue fever cases. They also perceived that the app was able to improve their knowledge, attitudes, and awareness of dengue fever. Although participants were positive toward the app, some issues were also raised: the availability of the app on iOS (iPhone) mobile phones only, language option limitations, the need for a within-app user’s guide (eg, pop-ups or labels), and polishing of the app’s technical and functional details (eg, buttons and gestures). Some suggestions were also given by the participants: the use of predictive surveillance, inclusion of other mosquito-borne diseases in the real-time reporting and mapping feature, and use of games to increase usability and engagement among users.

We have only managed to do minimal revisions (simpler log-in and sign-up pages and pin legend in the map screen) in the design of the app for an immediate trial and assessment in the Philippines.

We have developed and designed a mobile app, Mozzify, which obtained excellent acceptability and ratings (mean scores ≥4.0 out of 5) based on the MARS subscales among health experts and researchers and the general public, which indicate that it is ready for another trial among a larger population in the Philippines. It may be a promising integrated strategic health intervention system for reporting and mapping dengue fever cases; increasing awareness, improving knowledge, and changing attitudes about dengue fever; and disseminating and sharing information on dengue fever among the general population and health experts and for the knowledge on how to prevent dengue fever to be successfully translated to practice.

We have started to collect data on the longitudinal spatial analysis of dengue fever hotspots in the Philippines as a provision for a predictive surveillance feature, and the inclusion of other mosquito-borne diseases in the reporting and mapping system in the future. Thus, we also plan to design an alert system on the map that would warn users when they enter or when they are near a barangay/village or area with a high incidence of dengue fever cases and mosquito abundance. We are also working on developing an Android version of the app, more language options, and the possible inclusion of games to increase usability and engagement among users in the future. Our aim is that Mozzify will address the lack of available apps for the control and prevention of dengue fever not only in the Philippines but also in other countries with dengue fever and other mosquito-borne diseases worldwide.

## Appendix

Multimedia Appendix 1 Mozzify's user guide.

Multimedia Appendix 2 MARS form.

## Abbreviations

API: Application Programming Interface
COMBI: Communication for Behavioral Impact
MARS: Mobile Application Rating Scale
