# Supplementary material for: An Integrated mHealth App for Dengue Reporting and Mapping, Health Communication, and Behavior Modification: Development and Assessment of Mozzify
Source: JMIR Form Res. 2020 Jan 8;4(1):e16424. doi: 10.2196/16424 (PMC6996774; doi:10.2196/16424)
Supplement: Multimedia Appendix 1 [file formative_v4i1e16424_app1.pdf]

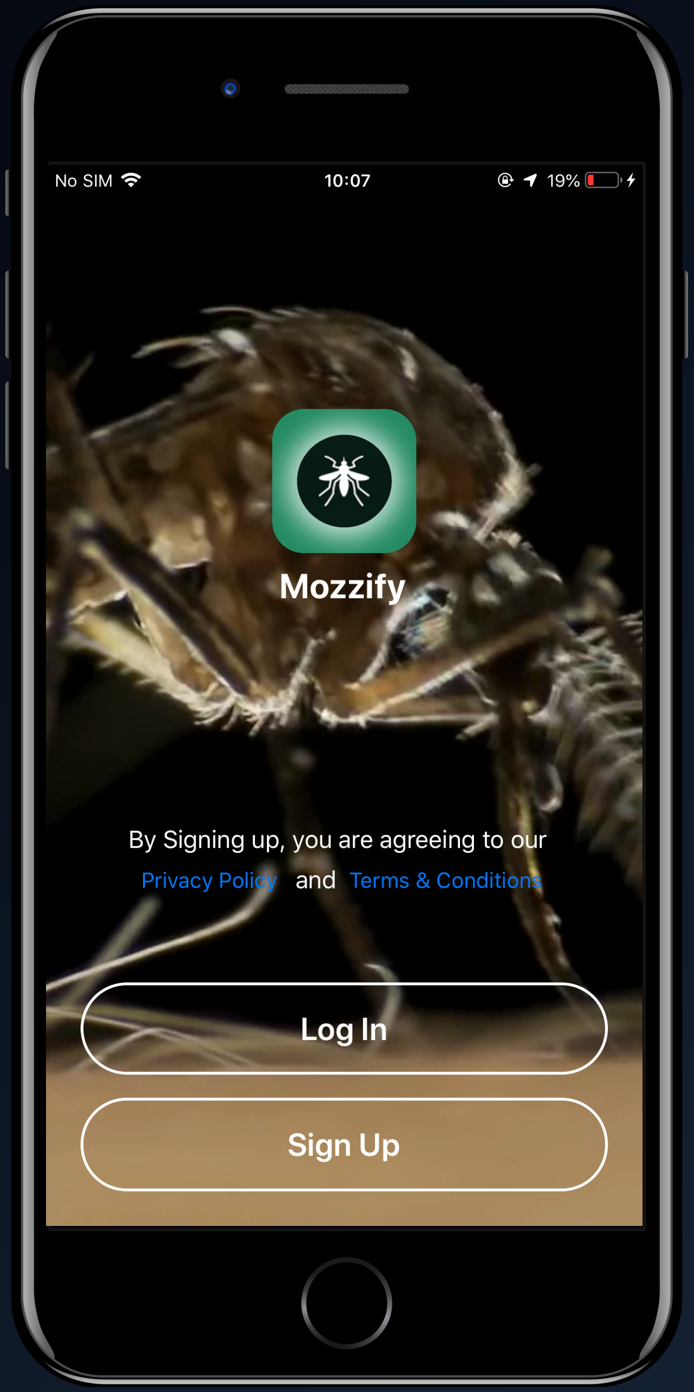

Mozzify

User's Guide

Mozzify User Guide. June 2019 version 1.0

All rights reserved. No part of this publication may be produced or transmitted in any form or by any means, including photocopying and recording, without seeking the permission of the developer. For inquiries, email herbuelavonralphdane@gmail.com

Contents

1.Introduction
- 1.1 Mozzify Mobile Application....3
- 1.2 Main Features....4
- 1.3 Privacy Policy 5
- 1.3 Terms and Conditions 9

2. Agreements
- Privacy Policy and Terms & Conditions....12

3. Sign-Up....13

4. Login 14

5. Map....13
- 5.1 Buttons and Gestures....15
- 5.2 Map Feature Views....16
- 5.3 Adding map pins (probable and mosquito bites)....17
- 5.4 Adding Image Attachments....18
- 5.5 Downloading Offline Map....19
- 5.6 Synchronizing & Deleting Offline Map....20

6. Profile
- 6.1 Profile....21
- 6.2 Signs and Symptoms & Hospital Directions....22
- 6.3 Clinical Data....23
- 6.4. PDF Collection....24

7. News and Timeline 25

8. Video....26

9. To Do
- 9.1 Transporting Preventive Practices to Reminders....27
- 9.2 Customizing Reminders....28
- 9.3 Adding New Practice, Completing and Deleting Practices....29

10. References....30

All rights reserved. No part of this publication may be produced or transmitted in any form or by any means, including photocopying and recording, without seeking the permission of the developer. For inquiries, email herbuelavonralphdane@gmail.com

1.1. Mozzify Mobile Application

Application Name: Mozzify

Version: 1

Platform: iOS iPhone (Apple)

Device Requirements: iPhone (iOS 11.0 and above)

Affiliations: Department of Civil and Environmental Engineering, Ehime University, Japan

Cost/In-App Purchase/Free: Free

Advertising: None

Brief description: This is an app that shows Dengue Fever (DF) cases and mosquito bite reports to identify DF hotspots. This has also a DF symptoms checker and hospital directions features. This informs users about DF by videos, news and websites of local and international health agencies. Users can also share their concerns and questions on DF using this app. Lastly, the app aims to help users to practice measures against DF using the Reminders feature.

Target/s: Physical health, Behavior change

Theoretical background/ Strategies: Assessment, Feedback, Information/Education, Monitoring/Tracking, Advice/ tips/Strategies/Skills training

Developer: Von Ralph Dane M. Herbuela (Academic, Non-Commercial)

Email: herbuelavonralphdane@gmail.com

Contact number: +81- 080-3925-2523

All rights reserved. No part of this publication may be produced or transmitted in any form or by any means, including photocopying and recording, without seeking the permission of the developer. For inquiries, email herbuelavonralphdane@gmail.com

1.2. Main Features

1. Case Reporting to identify Dengue Hotspots using ArcGIS Map

a. Case reporting. The main feature of this application is the use of data from ArcGIS. The map shows the prevalence of probable (User-reported), confirmed Dengue fever (DF) (Hospital-reported) cases and mosquito bites (User-reported). Probable cases are in blue pins (after a user shows symptoms of DF by checking the symptoms in the app) while confirmed DF cases are in red pins. Orange pins are mosquito bite reports from users.

b. DF Hotspots. Users will be able to see the prevalence (dark-colored) of probable and confirmed DF cases and mosquito bites in the map.

2. Symptoms Checker and Hospital Directions

a. User Profile. Users can make a profile which include personal information (e.g. age, gender, address, etc. and health history information (e.g. DF history, family DF history, medical conditions, DF vaccine etc.).

b. Signs and Symptoms. Users who manifest DF signs and symptoms can check the list of DF warning signs and symptoms. The app will detect if the warning signs will require the user to seek medical assessment by a physician in a hospital.

c. Hospital Directions. If the user qualifies as a probable DF patient, the app will alert him/her to go to the nearest hospital that caters DF cases (including those hospitals that cater Dengvaxia-vaccinated individuals) by showing directions.

3. Education and information Sharing

a. Educational Videos. Users can watch videos on DF virus, symptoms, diagnosis and treatment

b. News about latest issues on DF and other mosquito-borne diseases.

c. Websites of international and local health agencies (e.g. WHO and Department of Health).

d. Timeline: shows posts of events, concerns, and questions on DF among users.

4. Behavior Modification (Preventive practices against DF)

Reminder Alerts. (within device) Another function of this feature is the Reminder alerts program based from the COMBI (Communication for Behavioral Intervention) on the preventive practices against DF. This is expected to develop or improve users' behavior on the practice of preventive measures against DF.

All rights reserved. No part of this publication may be produced or transmitted in any form or by any means, including photocopying and recording, without seeking the permission of the developer. For inquiries, email herbuelavonralphdane@gmail.com

1.3. Privacy Policy

Effective date: June 17, 2019

Mozzify ("us", "we", or "our") operates the Mozzify mobile application (hereinafter referred to as the "Service").

This page informs you of our policies regarding the collection, use and disclosure of personal data when you use our Service and the choices you have associated with that data.

We use your data to provide and improve the Service. By using the Service, you agree to the collection and use of information in accordance with this policy. Unless otherwise defined in this Privacy Policy, the terms used in this Privacy Policy have the same meanings as in our Terms and Conditions.

Definitions

Service

Service is the Mozzify mobile application operated by Mozzify

Personal Data

Personal Data means data about a living individual who can be identified from those data (or from those and other information either in our possession or likely to come into our possession).

Usage Data

Usage Data is data collected automatically either generated by the use of the Service or from the Service infrastructure itself (for example, the duration of a page visit).

Cookies

Cookies are small files stored on your device (computer or mobile device).

Information Collection and Use

We collect several different types of information for various purposes to provide and improve our Service to you.

Types of Data Collected

Personal Data

While using our Service, we may ask you to provide us with certain personally identifiable information that can be used to contact or identify you ("Personal Data"). Personally identifiable information may include, but is not limited to:

- Email address
- First name and last name
- Phone number
- Address, State, Province, ZIP/Postal code, City

All rights reserved. No part of this publication may be produced or transmitted in any form or by any means, including photocopying and recording, without seeking the permission of the developer. For inquiries, email herbuelavonralphdane@gmail.com

- Cookies and Usage Data

Usage Data

When you access the Service with a mobile device, we may collect certain information automatically, including, but not limited to, the type of mobile device you use, your mobile device unique ID, the IP address of your mobile device, your mobile operating system, the type of mobile Internet browser you use, unique device identifiers and other diagnostic data ("Usage Data").

Location Data

We may use and store information about your location if you give us permission to do so ("Location Data"). We use this data to provide features of our Service, to improve and customise our Service.

You can enable or disable location services when you use our Service at any time by way of your device settings.

Tracking Cookies Data

We use cookies and similar tracking technologies to track the activity on our Service and we hold certain information.

Cookies are files with a small amount of data which may include an anonymous unique identifier. Cookies are sent to your browser from a website and stored on your device. Other tracking technologies are also used such as beacons, tags and scripts to collect and track information and to improve and analyse our Service.

You can instruct your browser to refuse all cookies or to indicate when a cookie is being sent. However, if you do not accept cookies, you may not be able to use some portions of our Service. Examples of Cookies we use:

- Session Cookies. We use Session Cookies to operate our Service.
- Preference Cookies. We use Preference Cookies to remember your preferences and various settings.
- Security Cookies. We use Security Cookies for security purposes.

Use of Data

Mozzify uses the collected data for various purposes:

- To provide and maintain our Service
- To notify you about changes to our Service
- To allow you to participate in interactive features of our Service when you choose to do so
- To provide customer support
- To gather analysis or valuable information so that we can improve our Service
- To monitor the usage of our Service
- To detect, prevent and address technical issues

All rights reserved. No part of this publication may be produced or transmitted in any form or by any means, including photocopying and recording, without seeking the permission of the developer. For inquiries, email herbuelavonralphdane@gmail.com

Transfer of Data

Your information, including Personal Data, may be transferred to - and maintained on - computers located outside of your state, province, country or other governmental jurisdiction where the data protection laws may differ from those of your jurisdiction.

If you are located outside Japan and choose to provide information to us, please note that we transfer the data, including Personal Data, to Japan and process it there.

Your consent to this Privacy Policy followed by your submission of such information represents your agreement to that transfer.

Mozzify will take all the steps reasonably necessary to ensure that your data is treated securely and in accordance with this Privacy Policy and no transfer of your Personal Data will take place to an organisation or a country unless there are adequate controls in place including the security of your data and other personal information.

Disclosure of Data

Legal Requirements

Mozzify may disclose your Personal Data in the good faith belief that such action is necessary to:

- To comply with a legal obligation
- To protect and defend the rights or property of Mozzify
- To prevent or investigate possible wrongdoing in connection with the Service
- To protect the personal safety of users of the Service or the public
- To protect against legal liability

Security of Data

The security of your data is important to us but remember that no method of transmission over the Internet or method of electronic storage is 100% secure. While we strive to use commercially acceptable means to protect your Personal Data, we cannot guarantee its absolute security.

Service Providers

We may employ third party companies and individuals to facilitate our Service ("Service Providers"), provide the Service on our behalf, perform Service-related services or assist us in analysing how our Service is used.

These third parties have access to your Personal Data only to perform these tasks on our behalf and are obligated not to disclose or use it for any other purpose.

Analytics

We may use third-party Service Providers to monitor and analyse the use of our Service.

Firebase

Firebase is an analytics service provided by Google Inc.

You may opt-out of certain Firebase features through your mobile device settings, such as your device advertising settings or by following the instructions provided by Google in their Privacy Policy: https://policies.google.com/privacy?hl=en

All rights reserved. No part of this publication may be produced or transmitted in any form or by any means, including photocopying and recording, without seeking the permission of the developer. For inquiries, email herbuelavonralphdane@gmail.com

We also encourage you to review the Google's policy for safeguarding your data: https://support.google.com/analytics/answer/6004245.
For more information on what type of information Firebase collects, please visit the Google Privacy Terms web page: https://policies.google.com/privacy?hl=en

Links to Other Sites

Our Service may contain links to other sites that are not operated by us. If you click a third party link, you will be directed to that third party's site. We strongly advise you to review the Privacy Policy of every site you visit.

We have no control over and assume no responsibility for the content, privacy policies or practices of any third party sites or services.

Children's Privacy

Our Service does not address anyone under the age of 18 ("Children").
We do not knowingly collect personally identifiable information from anyone under the age of 18. If you are a parent or guardian and you are aware that your Child has provided us with Personal Data, please contact us. If we become aware that we have collected Personal Data from children without verification of parental consent, we take steps to remove that information from our servers.

Changes to This Privacy Policy

We may update our Privacy Policy from time to time. We will notify you of any changes by posting the new Privacy Policy on this page.

We will let you know via email and/or a prominent notice on our Service, prior to the change becoming effective and update the "effective date" at the top of this Privacy Policy.

You are advised to review this Privacy Policy periodically for any changes. Changes to this Privacy Policy are effective when they are posted on this page.

Contact Us

If you have any questions about this Privacy Policy, please contact us:

- By email: herbuelavonralphdane@gmail.com
- By phone number: +818039252523

This Privacy policy was generated using Termsfeed.com services:
https://www.termsfeed.com/privacy-policy/3df700ca09adc7250a48b7abfd83d081

All rights reserved. No part of this publication may be produced or transmitted in any form or by any means, including photocopying and recording, without seeking the permission of the developer. For inquiries, email herbuelavonralphdane@gmail.com

1.4. Terms and Conditions

Last updated: June 17, 2019

These Terms and Conditions ("Terms", "Terms and Conditions") govern your relationship with Mozzify mobile application (the "Service") operated by Mozzify ("us", "we", or "our"). Please read these Terms and Conditions carefully before using our Mozzify mobile application (the "Service").

Your access to and use of the Service is conditioned on your acceptance of and compliance with these Terms. These Terms apply to all visitors, users and others who access or use the Service. By accessing or using the Service you agree to be bound by these Terms. If you disagree with any part of the terms then you may not access the Service.

Content

Our Service allows you to post, link, store, share and otherwise make available certain information, text, graphics, videos, or other material ("Content"). You are responsible for the Content that you post to the Service, including its legality, reliability, and appropriateness.

By posting Content to the Service, you grant us the right and license to use, modify, publicly perform, publicly display, reproduce, and distribute such Content on and through the Service. You retain any and all of your rights to any Content you submit, post or display on or through the Service and you are responsible for protecting those rights. You agree that this license includes the right for us to make your Content available to other users of the Service, who may also use your Content subject to these Terms.

You represent and warrant that: (i) the Content is yours (you own it) or you have the right to use it and grant us the rights and license as provided in these Terms, and (ii) the posting of your Content on or through the Service does not violate the privacy rights, publicity rights, copyrights, contract rights or any other rights of any person.

Accounts

When you create an account with us, you must provide us information that is accurate, complete, and current at all times. Failure to do so constitutes a breach of the Terms, which may result in immediate termination of your account on our Service.

You are responsible for safeguarding the password that you use to access the Service and for any activities or actions under your password, whether your password is with our Service or a third-party service.

You agree not to disclose your password to any third party. You must notify us immediately upon becoming aware of any breach of security or unauthorized use of your account.

You may not use as a username the name of another person or entity or that is not lawfully available for use, a name or trade mark that is subject to any rights of another person or entity

All rights reserved. No part of this publication may be produced or transmitted in any form or by any means, including photocopying and recording, without seeking the permission of the developer. For inquiries, email herbuelavonralphdane@gmail.com

other than you without appropriate authorization, or a name that is otherwise offensive, vulgar or obscene.

Links To Other Web Sites

Our Service may contain links to third-party web sites or services that are not owned or controlled by Mozzify.

Mozzify has no control over, and assumes no responsibility for, the content, privacy policies, or practices of any third party web sites or services. You further acknowledge and agree that Mozzify shall not be responsible or liable, directly or indirectly, for any damage or loss caused or alleged to be caused by or in connection with use of or reliance on any such content, goods or services available on or through any such web sites or services.

We strongly advise you to read the terms and conditions and privacy policies of any third-party web sites or services that you visit.

Termination

We may terminate or suspend your account immediately, without prior notice or liability, for any reason whatsoever, including without limitation if you breach the Terms.

Upon termination, your right to use the Service will immediately cease. If you wish to terminate your account, you may simply discontinue using the Service.

Limitation Of Liability

In no event shall Mozzify, nor its directors, employees, partners, agents, suppliers, or affiliates, be liable for any indirect, incidental, special, consequential or punitive damages, including without limitation, loss of profits, data, use, goodwill, or other intangible losses, resulting from (i) your access to or use of or inability to access or use the Service; (ii) any conduct or content of any third party on the Service; (iii) any content obtained from the Service; and (iv) unauthorized access, use or alteration of your transmissions or content, whether based on warranty, contract, tort (including negligence) or any other legal theory, whether or not we have been informed of the possibility of such damage, and even if a remedy set forth herein is found to have failed of its essential purpose.

Disclaimer

Your use of the Service is at your sole risk. The Service is provided on an "AS IS" and "AS AVAILABLE" basis. The Service is provided without warranties of any kind, whether express or implied, including, but not limited to, implied warranties of merchantability, fitness for a particular purpose, non-infringement or course of performance.

Mozzify its subsidiaries, affiliates, and its licensors do not warrant that a) the Service will function uninterrupted, secure or available at any particular time or location; b) any errors or defects will be corrected; c) the Service is free of viruses or other harmful components; or d) the results of using the Service will meet your requirements.

All rights reserved. No part of this publication may be produced or transmitted in any form or by any means, including photocopying and recording, without seeking the permission of the developer. For inquiries, email herbuelavonralphdane@gmail.com

Governing Law

These Terms shall be governed and construed in accordance with the laws of Japan, without regard to its conflict of law provisions.

Our failure to enforce any right or provision of these Terms will not be considered a waiver of those rights. If any provision of these Terms is held to be invalid or unenforceable by a court, the remaining provisions of these Terms will remain in effect. These Terms constitute the entire agreement between us regarding our Service, and supersede and replace any prior agreements we might have between us regarding the Service.

Changes

We reserve the right, at our sole discretion, to modify or replace these Terms at any time. If a revision is material we will try to provide at least 30 days notice prior to any new terms taking effect. What constitutes a material change will be determined at our sole discretion.

By continuing to access or use our Service after those revisions become effective, you agree to be bound by the revised terms. If you do not agree to the new terms, please stop using the Service.

Contact Us

If you have any questions about these Terms, please contact us.

This Terms and Conditions was generated using Termsfeed.com services:

https://www.termsfeed.com/terms-conditions/b1319b1bdeea6f822a825a054e4d8b9f

All rights reserved. No part of this publication may be produced or transmitted in any form or by any means, including photocopying and recording, without seeking the permission of the developer. For inquiries, email herbuelavonralphdane@gmail.com

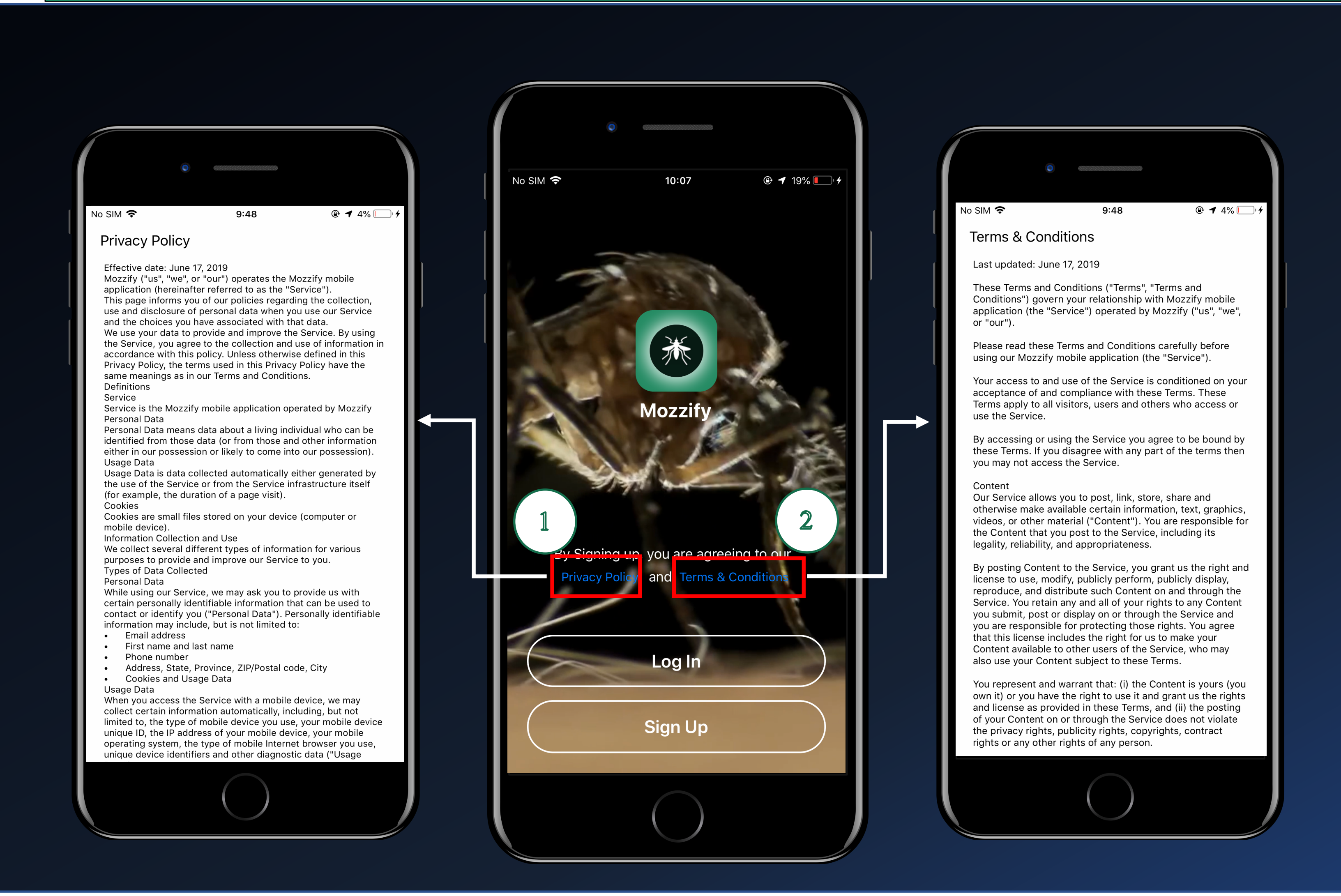

By signing-up, you are agreeing to our **Privacy Policy and Terms and Conditions**.

1. Click the ***Privacy Policy*** button to read the **Privacy Policy** statement. Scroll down the page and click the ***Mozzify icon*** button to go back to the main menu.

2. Click the ***Terms and Conditions*** button to read the **Terms & Conditions** statement. Scroll down the page and click the ***Mozzify icon*** button to go back to the main menu.

All rights reserved. No part of this publication may be produced or transmitted in any form or by any means, including photocopying and recording, without seeking the permission of the developer. For inquiries, email herbuelavonralphdane@gmail.com

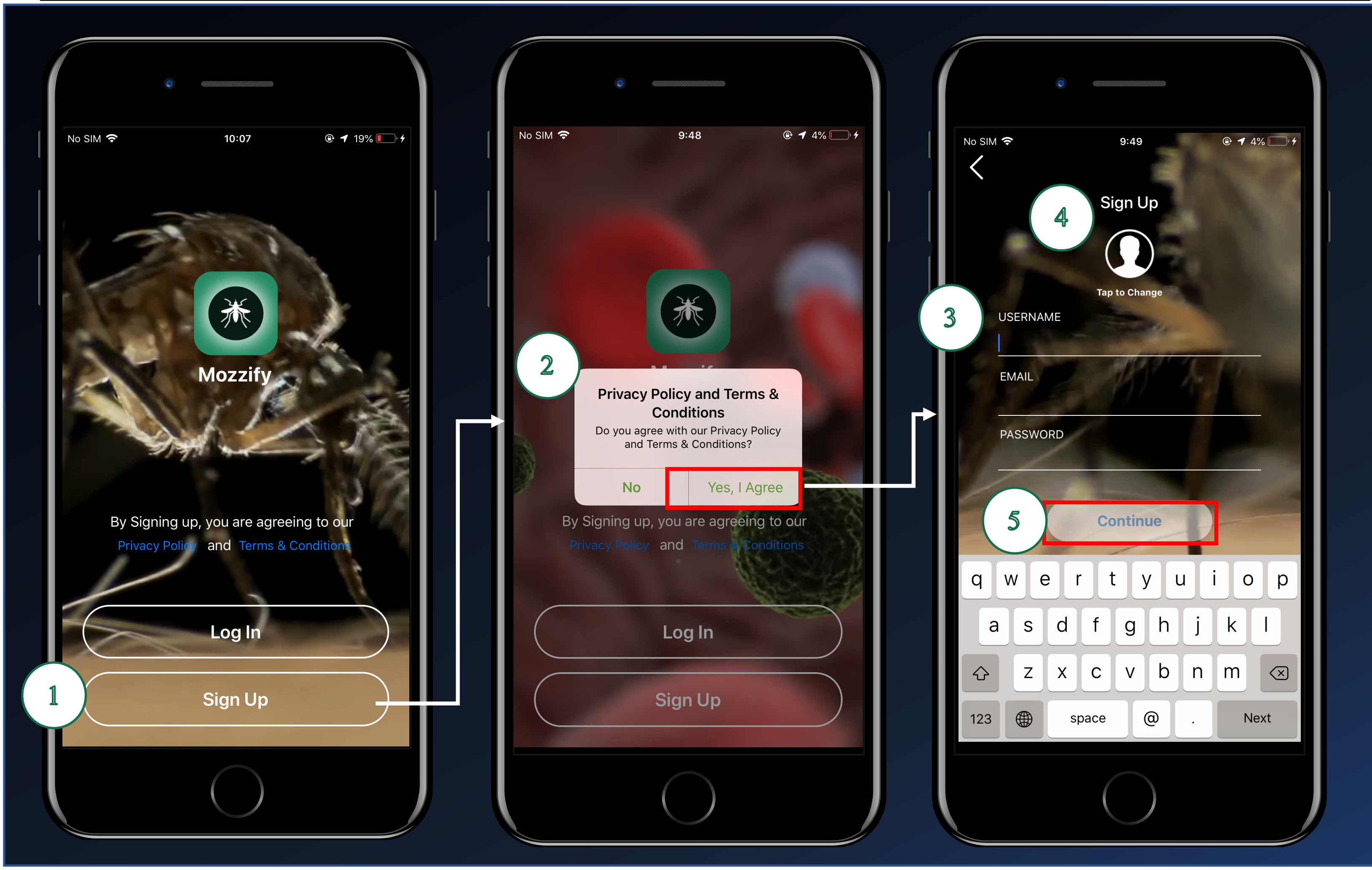

1. Click ***Sign Up*** button.

2. Click the ***Yes, I Agree*** button if you agree with the **Privacy Policy and Terms and Conditions.**

3. Enter your preferred **username**, **email address** and **password** in the text fields.

4. Click the 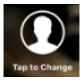 button to upload your profile picture. Then select an image from your photo gallery.

5. Click ***Continue*** button to sign-up. **Error** alert will pop-up if the email address was already registered.

All rights reserved. No part of this publication may be produced or transmitted in any form or by any means, including photocopying and recording, without seeking the permission of the developer. For inquiries, email herbuelavonralphdane@gmail.com

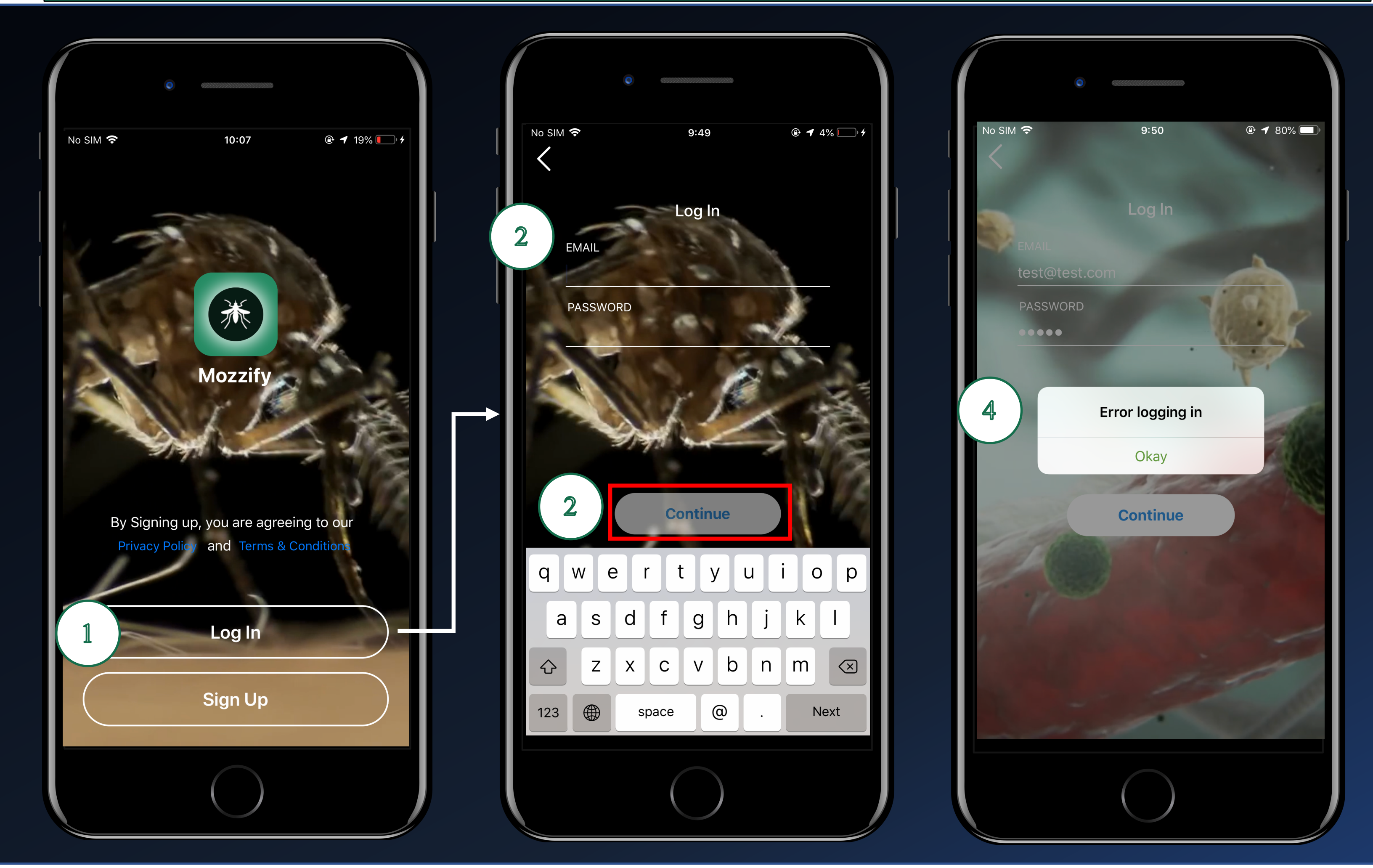

1. Click ***Log In*** button.

2. Enter the same **email address** and **password** used during Sign-up.

3. Click ***Continue*** button to log-in.

4. **Error** alert will pop-up if the **email address** and/or **password** your provided was/were wrong or not yet registered.

All rights reserved. No part of this publication may be produced or transmitted in any form or by any means, including photocopying and recording, without seeking the permission of the developer. For inquiries, email herbuelavonralphdane@gmail.com

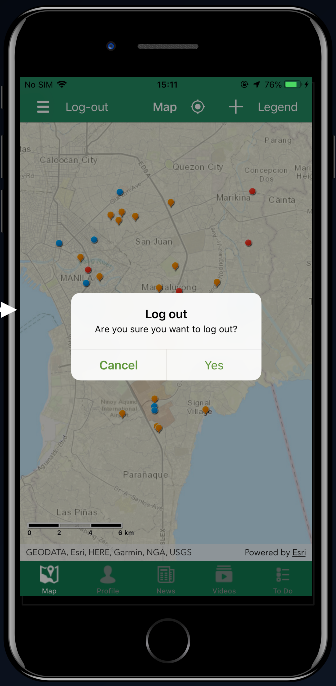

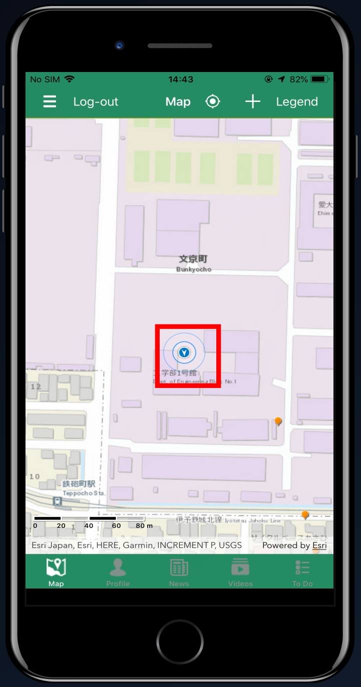

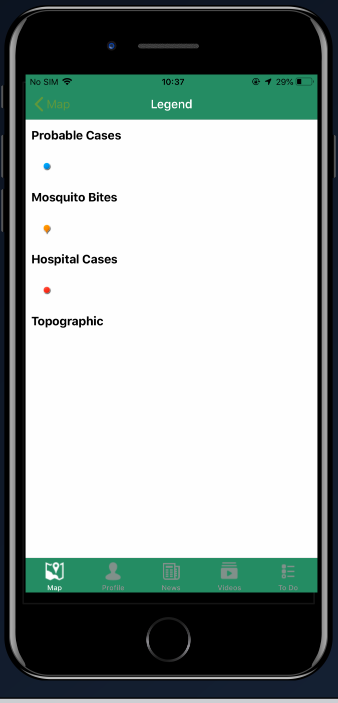

Navigation: Displays your current location

Legend: Displays a legend for a single layer in the map (and sub layers).

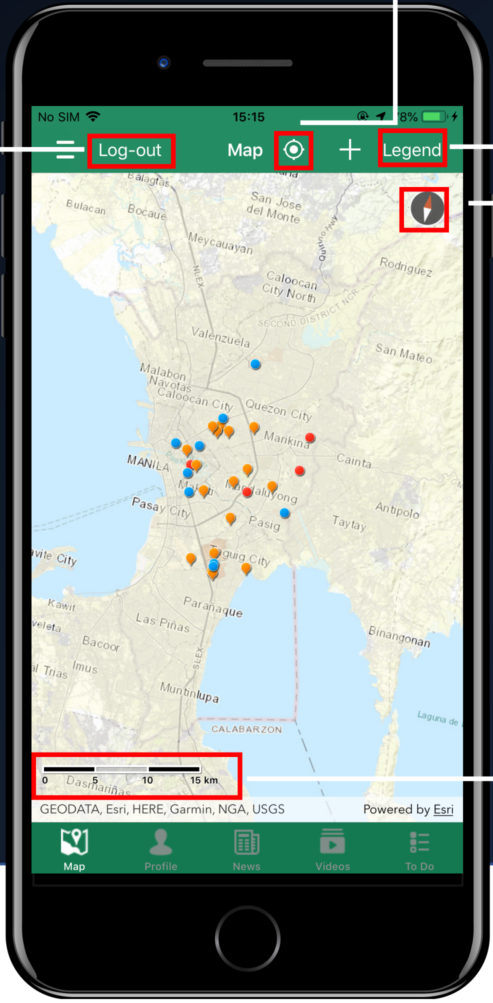

Compass: Shows a compass direction when the map is rotated. Auto-hides when the map points north up.

Scale line: Displays current scale reference

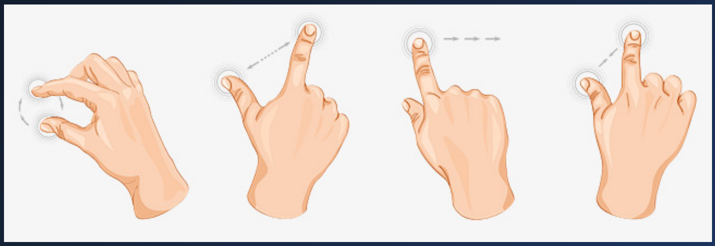

Map Gestures: Rotate, Zoom-In, Move, Zoom-Out

All rights reserved. No part of this publication may be produced or transmitted in any form or by any means, including photocopying and recording, without seeking the permission of the developer. For inquiries, email herbuelavonralphdane@gmail.com

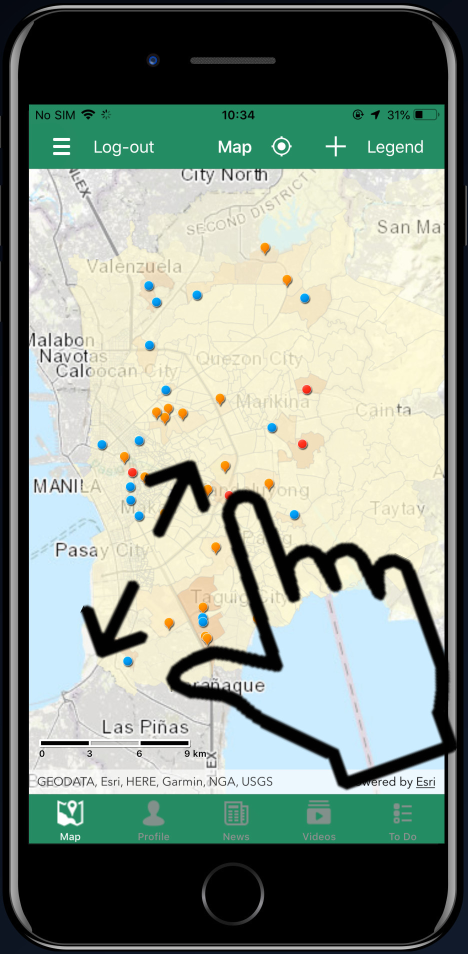

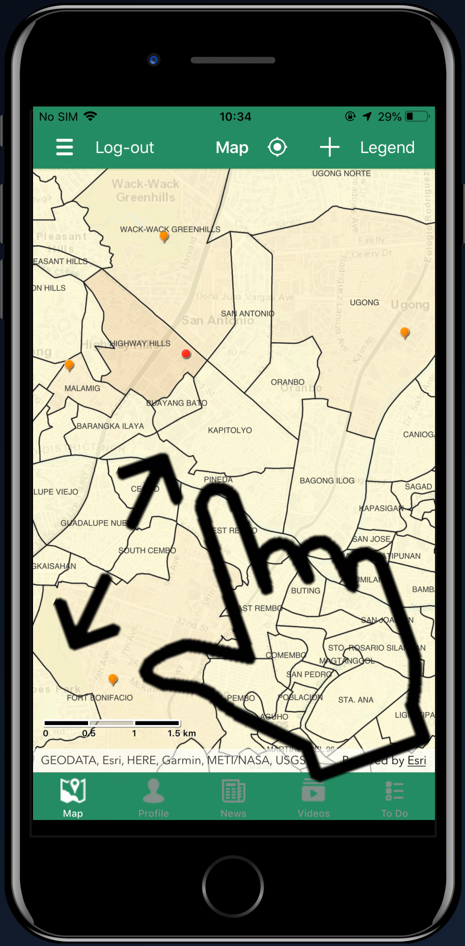

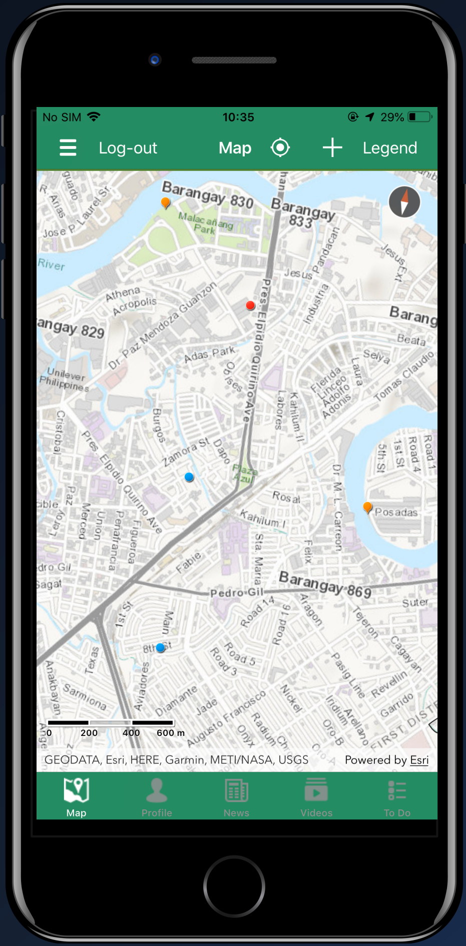

City Level:
9km in Scale line
You will see:

1. Map pins
2. Disease Hotspots (in brown)

Barangay (Village) Level:
1.5km in Scale line
You will see:

1. Map pins
2. Disease Hotspots (in brown)
3. Barangay (village) names and boundaries

Street Level:
600m in Scale line
You will see:

1. Map pins
2. Barangay (village) names
3. Street names
4. Houses and buildings
5. Land use (e.g. park, schools, etc.)

All rights reserved. No part of this publication may be produced or transmitted in any form or by any means, including photocopying and recording, without seeking the permission of the developer. For inquiries, email herbuelavonralphdane@gmail.com

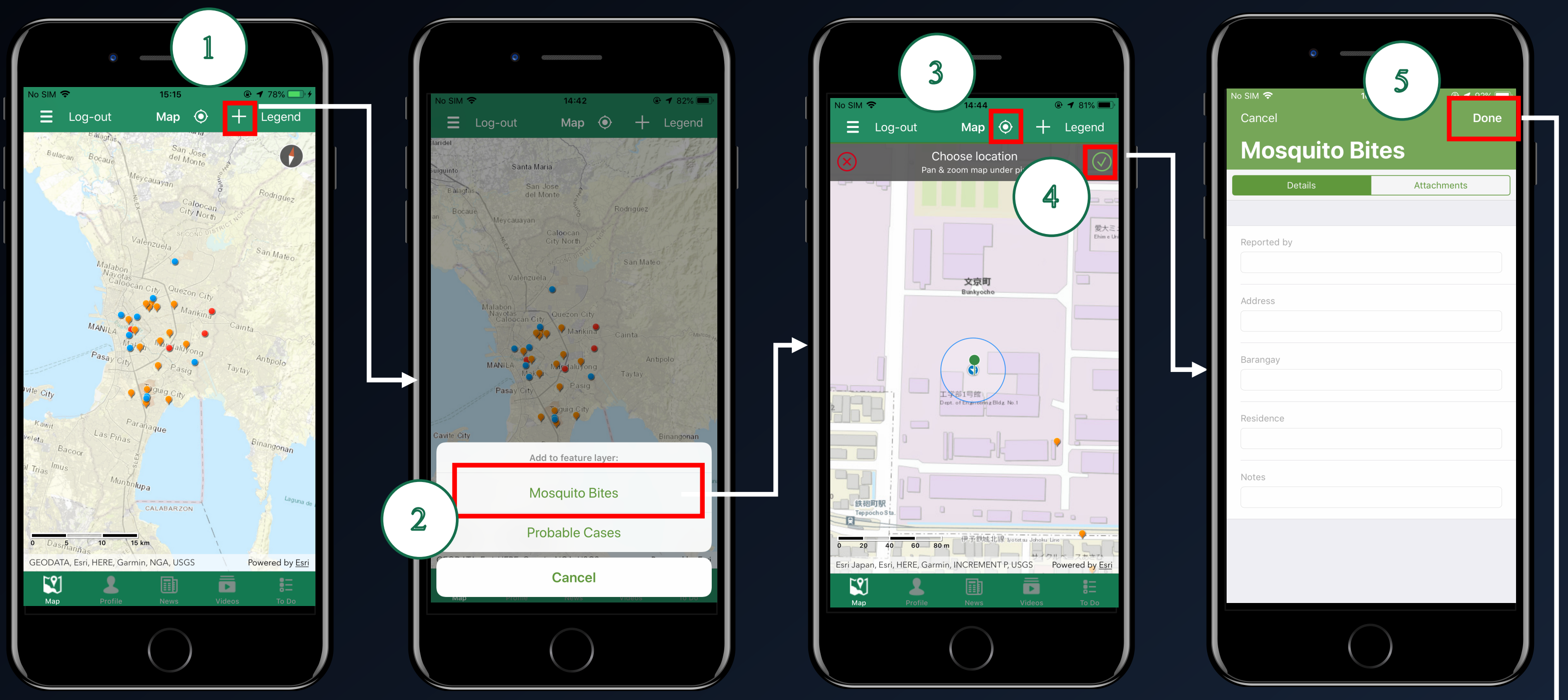

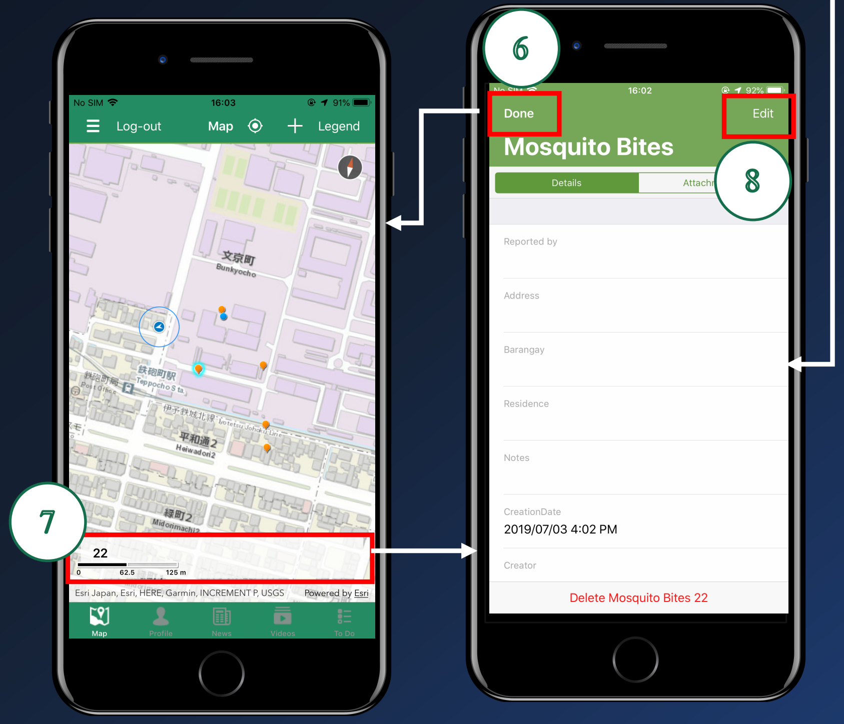

1. Click the + button to add map pins.

2. Click either ***Mosquito Bites*** or **Probable Cases** button.

3. Pan the **green pin** to the location you prefer or click the ***navigation*** button to automatically find your current location.

4. Click to pin the location. Fill-out the information needed.

5. Click ***Done*** button to save the information.

6. Click ***Done*** button to save the pin and to go back to the map.

7. Click the pop-up to show pin information.

<u>To Edit:</u>

7. Click the pop-up to show pin information.

8. Click ***Edit*** button and click ***Done*** button to save it.

All rights reserved. No part of this publication may be produced or transmitted in any form or by any means, including photocopying and recording, without seeking the permission of the developer. For inquiries, email herbuelavonralphdane@gmail.com

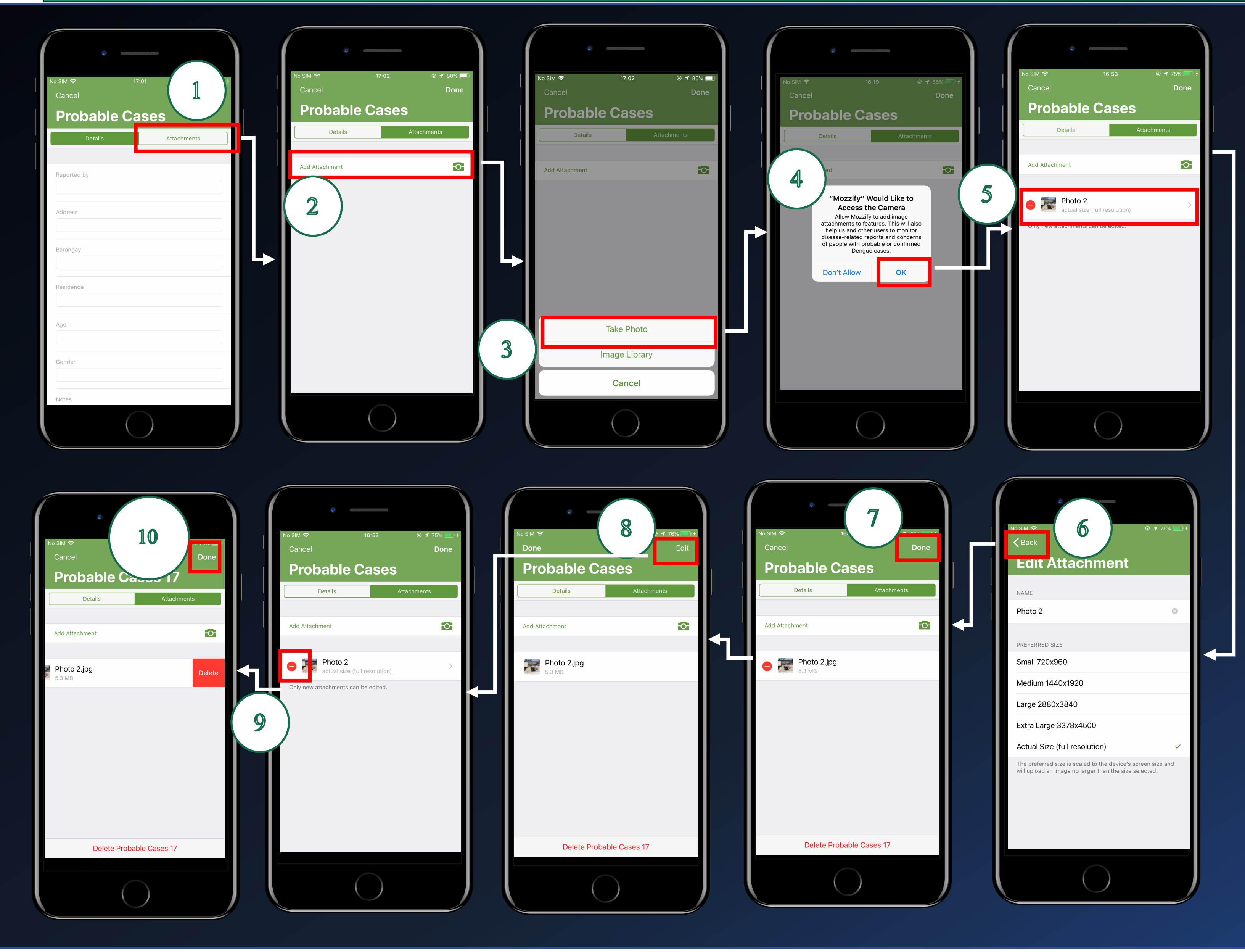

Refer to section **6.2. Adding map pins (probable and mosquito bites)** and follow the **steps 1 to 4.**

1. Click ***Attachments*** button.

2. Click ***Add Attachment*** button.

3. Click ***Take Photo*** or ***Image Library*** option.

4. Click OK to allow Mozzify access your **Camera** or **Image Library**.

5. Click the attachment to edit its **name** and choose **preferred size**.

6. Click ***Back*** button.

7. Click ***Done*** button to save the record.

<u>To Delete an image:</u>

8. Click ***Edit*** button.

9. Click – button and click ***Delete*** button

10. Click ***Done*** button to save and click ***Done.***

All rights reserved. No part of this publication may be produced or transmitted in any form or by any means, including photocopying and recording, without seeking the permission of the developer. For inquiries, email herbuelavonralphdane@gmail.com

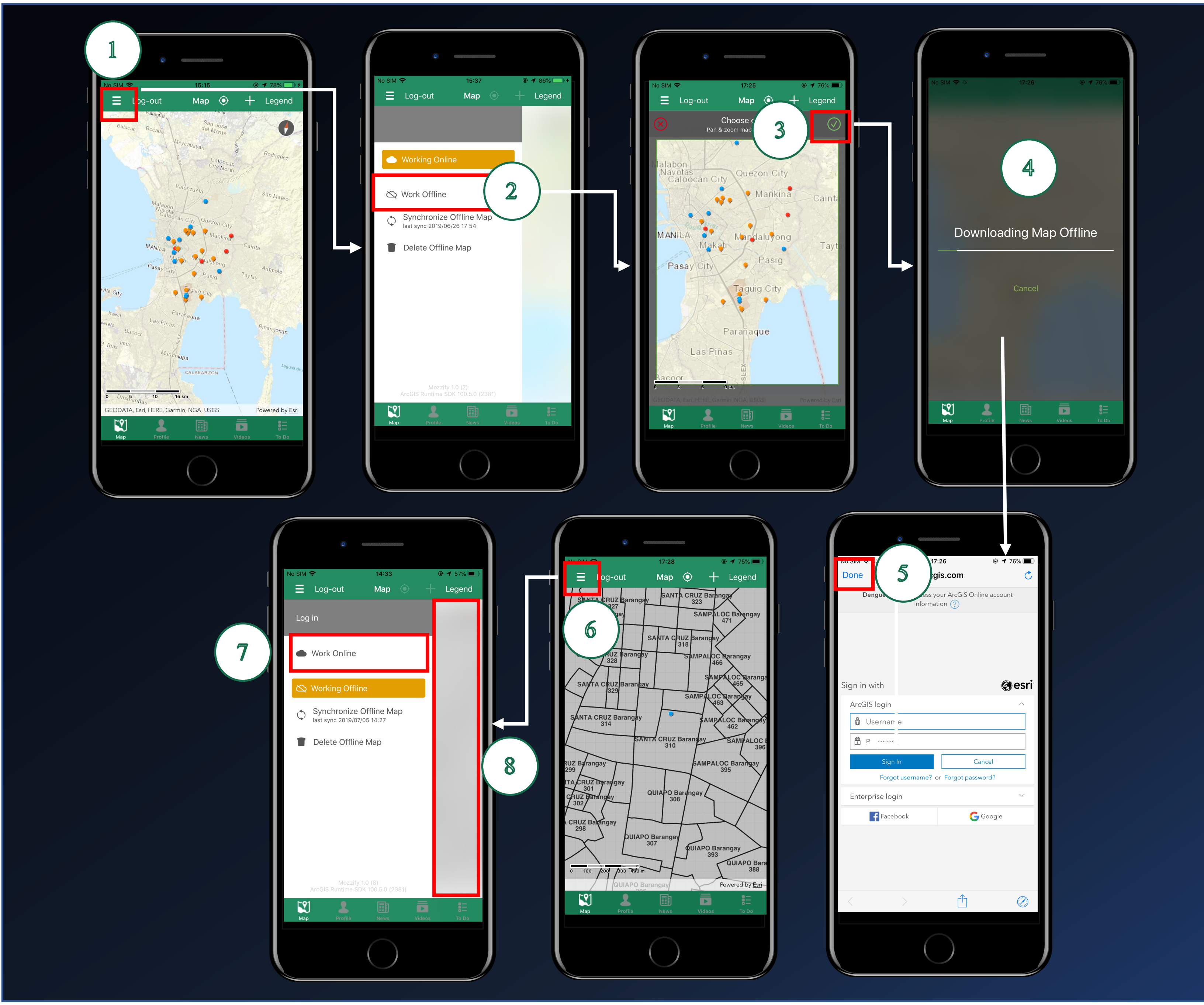

1. Click to show the drawer.

2. Click ***Work Offline*** button.

3. Zoom-in or pan the map to the location you prefer and click the ***green check*** button to download.

4. Download **Map Offline**.

5. Click ***Done*** (Do not enter username or password).

<u>To go back to Online Map:</u>

6. Click to show the drawer.

7. Click ***Work Online*** button.

8. Click to show online map

All rights reserved. No part of this publication may be produced or transmitted in any form or by any means, including photocopying and recording, without seeking the permission of the developer. For inquiries, email herbuelavonralphdane@gmail.com

5. Map

5.6. Synchronizing and Deleting Offline Map

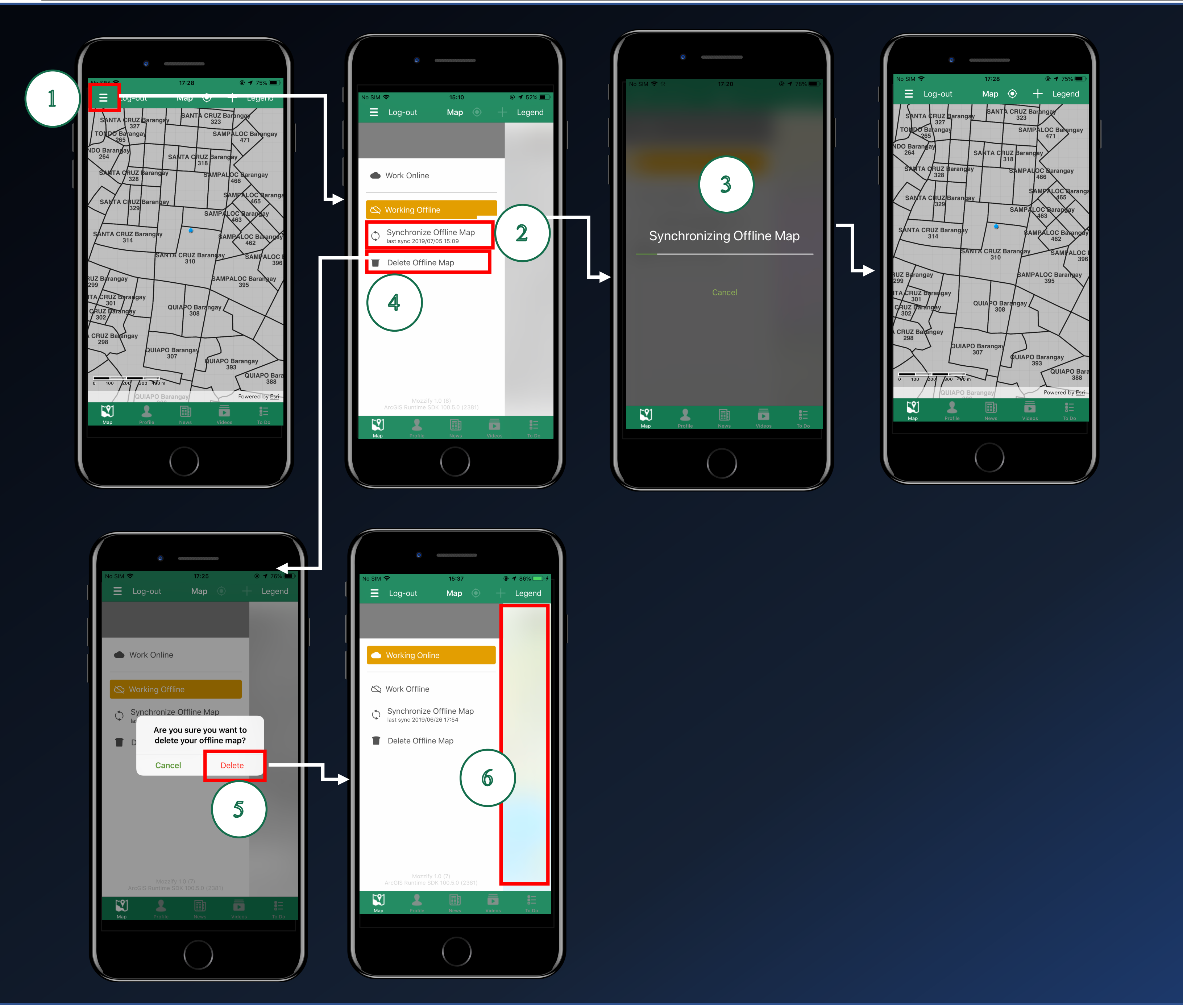

While in Offline Map:

1. Click to show the drawer.

2. Click ***Synchronize Offline Map*** button.

3. Synchronizing Offline Map.

<u>To Delete Offline Map:</u>

4. Click ***Delete Offline Map*** button.

5. Click **Delete** option in the alert.

6. Click to see the go back to **online map**.

All rights reserved. No part of this publication may be produced or transmitted in any form or by any means, including photocopying and recording, without seeking the permission of the developer. For inquiries, email herbuelavonralphdane@gmail.com

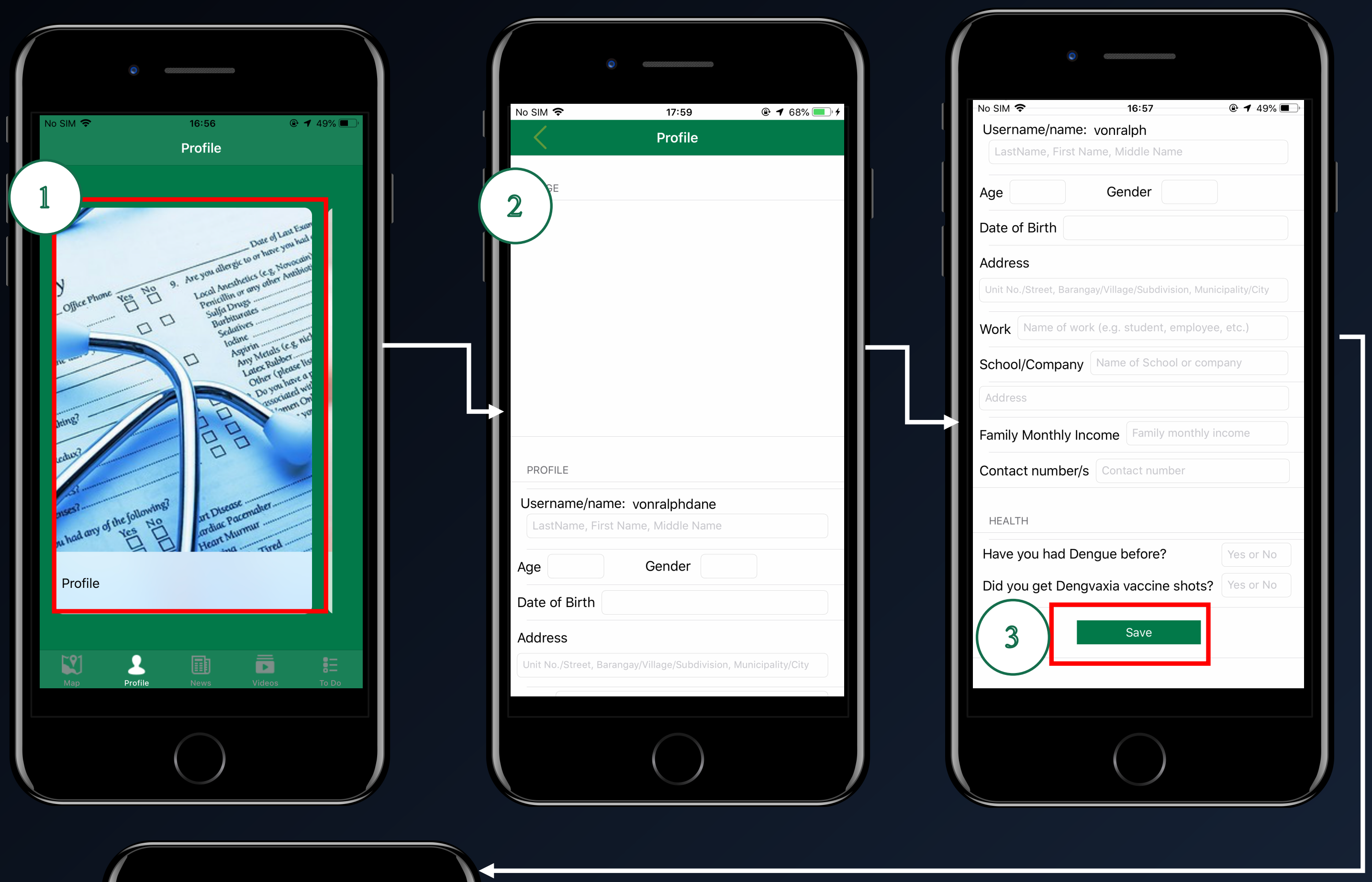

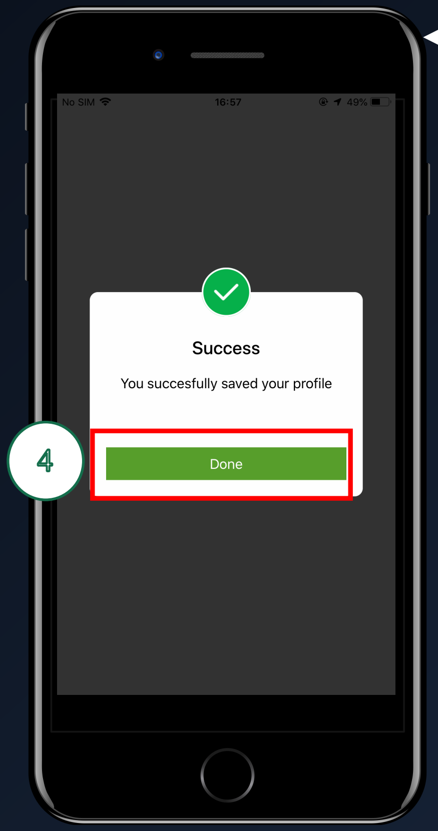

1. Click the **Profile** window.

2. Enter your information by clicking the text fields (optional).

3. Click ***Save*** button.

4. Click ***Done*** button in the **Success** alert.

The information that you will provide will be saved in a secure and private password-protected Firebase online database account. Please see section 1.1. **Privacy Policy** and 1.2. **Terms and Conditions** for more information.

All rights reserved. No part of this publication may be produced or transmitted in any form or by any means, including photocopying and recording, without seeking the permission of the developer. For inquiries, email herbuelavonralphdane@gmail.com

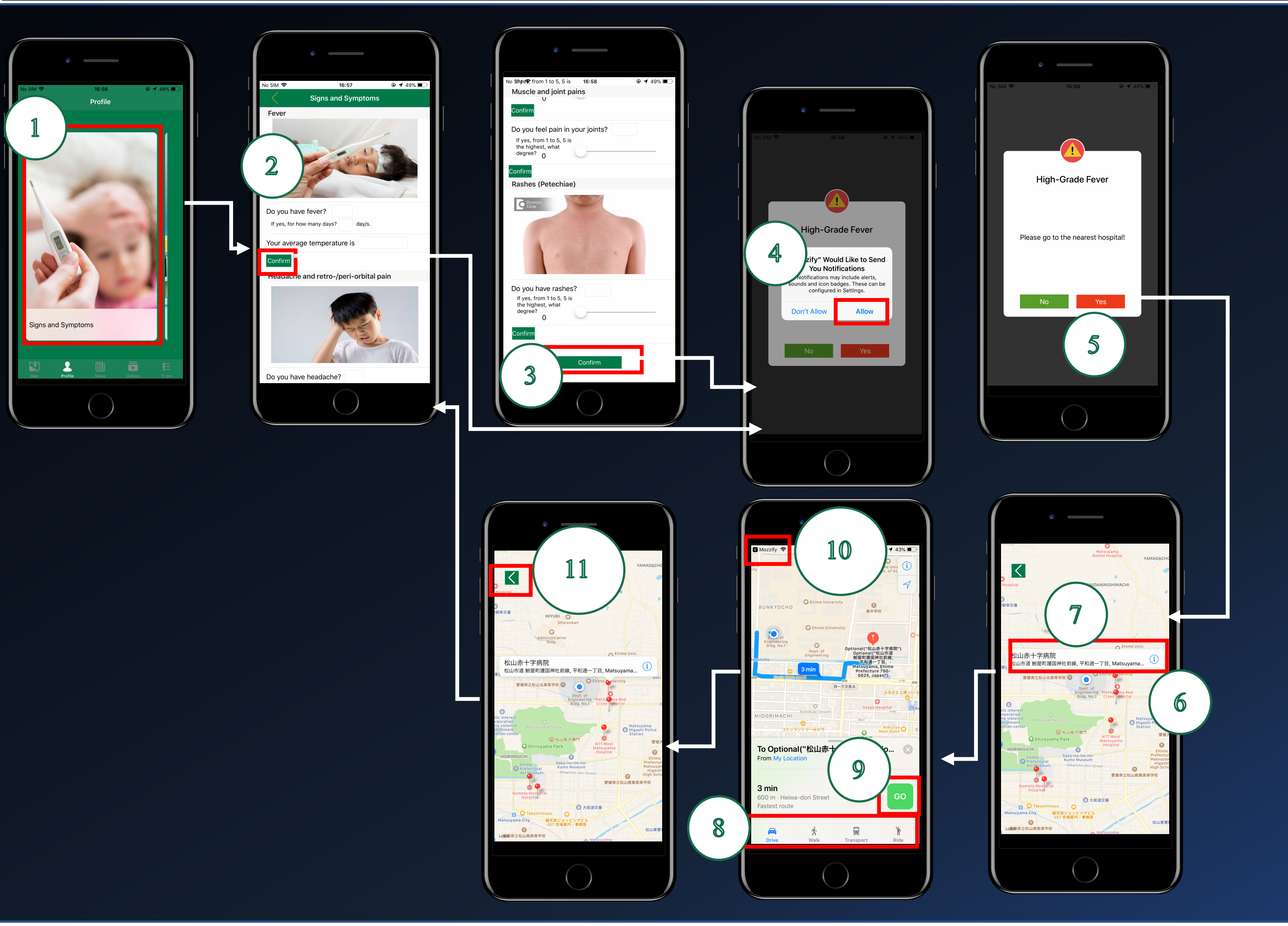

1. Click **Signs and Symptoms** window.

2. Answer all the questions and click the ***Confirm*** button in each symptom. If your condition requires immediate clinical assessment, the app will alert you to go to a nearest hospital.

3. Finish answering all the questions in all the symptoms and click ***Confirm***.

4. Click ***Allow*** button to send you notifications.

5. Click ***Yes*** button in the alert to show hospitals around you.

6. Click the **red** pin to show the hospital information.

7. Click to show directions.

8. Choose among the **options**.

9. Click ***GO*** button to start route.

10. Click the **Mozzify** in the upper leftmost part of the screen to go back.

11. Click ***back arrow*** button to go back.

All rights reserved. No part of this publication may be produced or transmitted in any form or by any means, including photocopying and recording, without seeking the permission of the developer. For inquiries, email herbuelavonralphdane@gmail.com

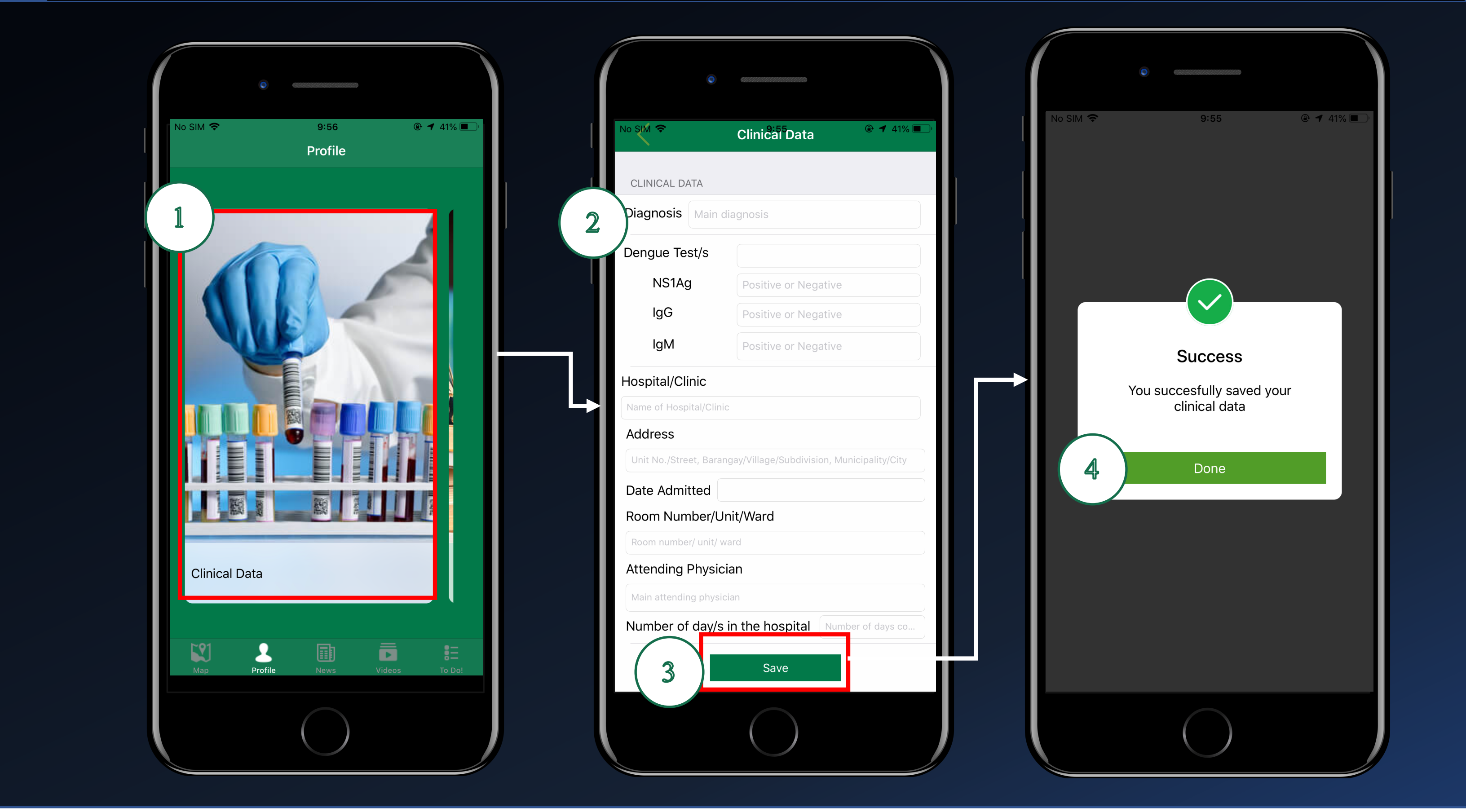

1. Click the **Clinical Data** window.

2. Enter your information by clicking the text fields (optional).

3. Click ***Save*** button.

4. Click ***Done*** button in the **Success** alert.

The information that you will provide will be saved in a secure and private password-protected Firebase online database account. Please see section 1.1. **Privacy Policy** and 1.2. **Terms and Conditions** for more information.

All rights reserved. No part of this publication may be produced or transmitted in any form or by any means, including photocopying and recording, without seeking the permission of the developer. For inquiries, email herbuelavonralphdane@gmail.com

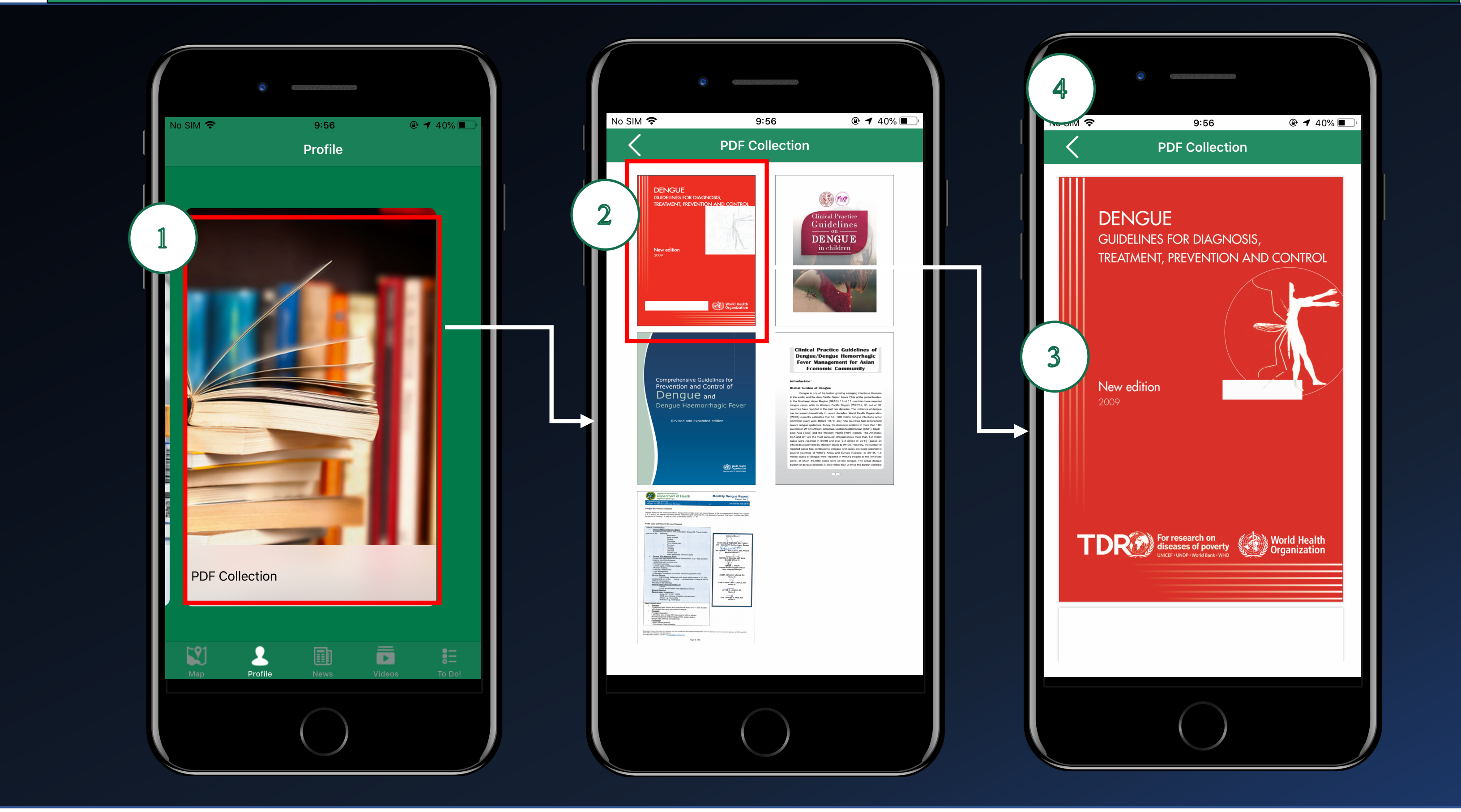

1. Click the **PDF Collection** window.

2. Click PDF icon to open.

3. Scroll down to turn pages, you can also zoom-in or zoom-out the pages.

4. Click ***back arrow*** button to go back.

All rights reserved. No part of this publication may be produced or transmitted in any form or by any means, including photocopying and recording, without seeking the permission of the developer. For inquiries, email herbuelavonralphdane@gmail.com

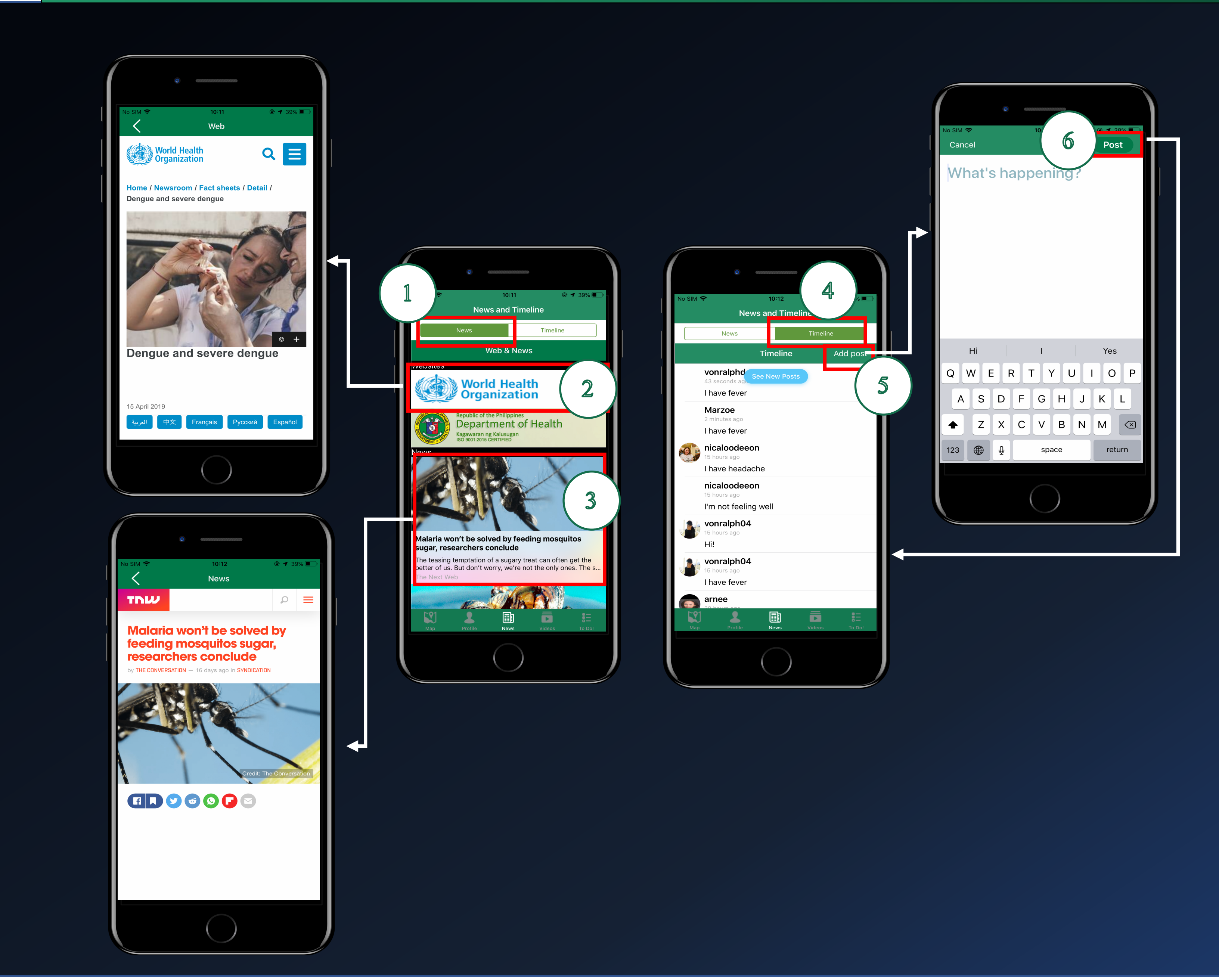

News

1. Click **News** tab button.

2. Click the WHO (or Department of Health) logo to go to their website.

3. Scroll down and click news boxes to see the latest news about mosquitoes, dengue fever, etc.

Timeline

4. Click ***Timeline*** button.

5. To add new post (present dengue-related events e.g.fogging, mosquito breeding sites, questions about symptoms and prevention, etc.]) click ***Add post*** button.

6. Click **Post** to post to timeline.

All rights reserved. No part of this publication may be produced or transmitted in any form or by any means, including photocopying and recording, without seeking the permission of the developer. For inquiries, email herbuelavonralphdane@gmail.com

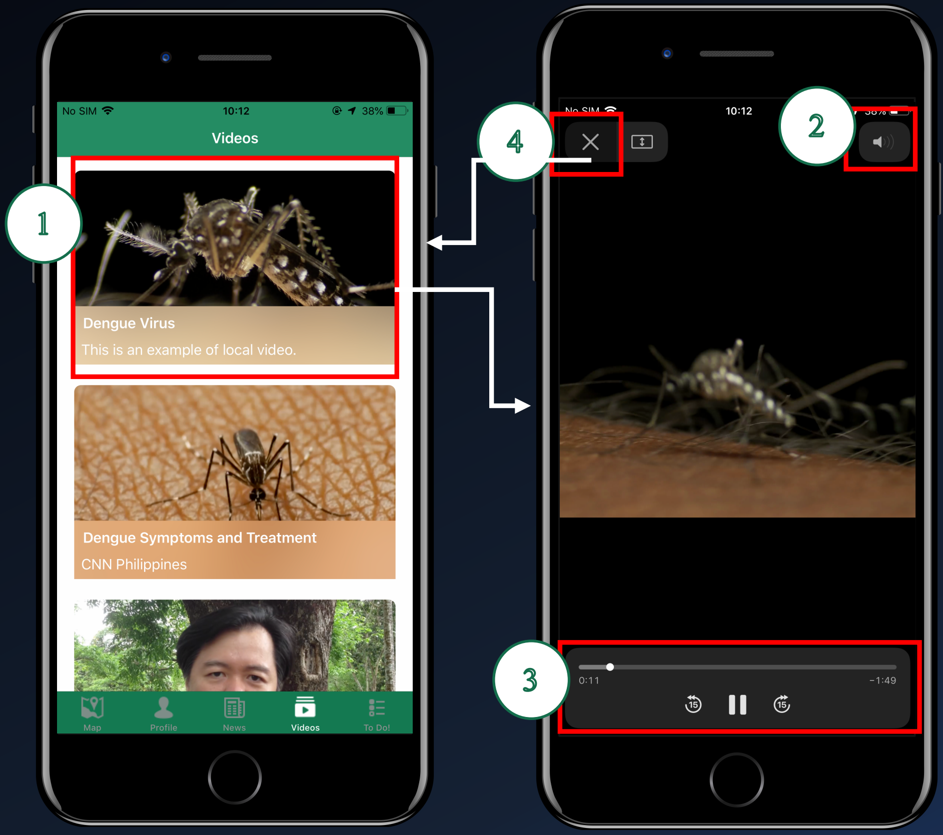

1. Scroll and click the **video icon image** button to play video.

2. Adjust **sound**.

3. Click ***play or pause*** buttons, and ***fast forward or back*** buttons to go back and forth the video.

4. Click ***x*** button or **drag down** the view to go back.

All rights reserved. No part of this publication may be produced or transmitted in any form or by any means, including photocopying and recording, without seeking the permission of the developer. For inquiries, email herbuelavonralphdane@gmail.com

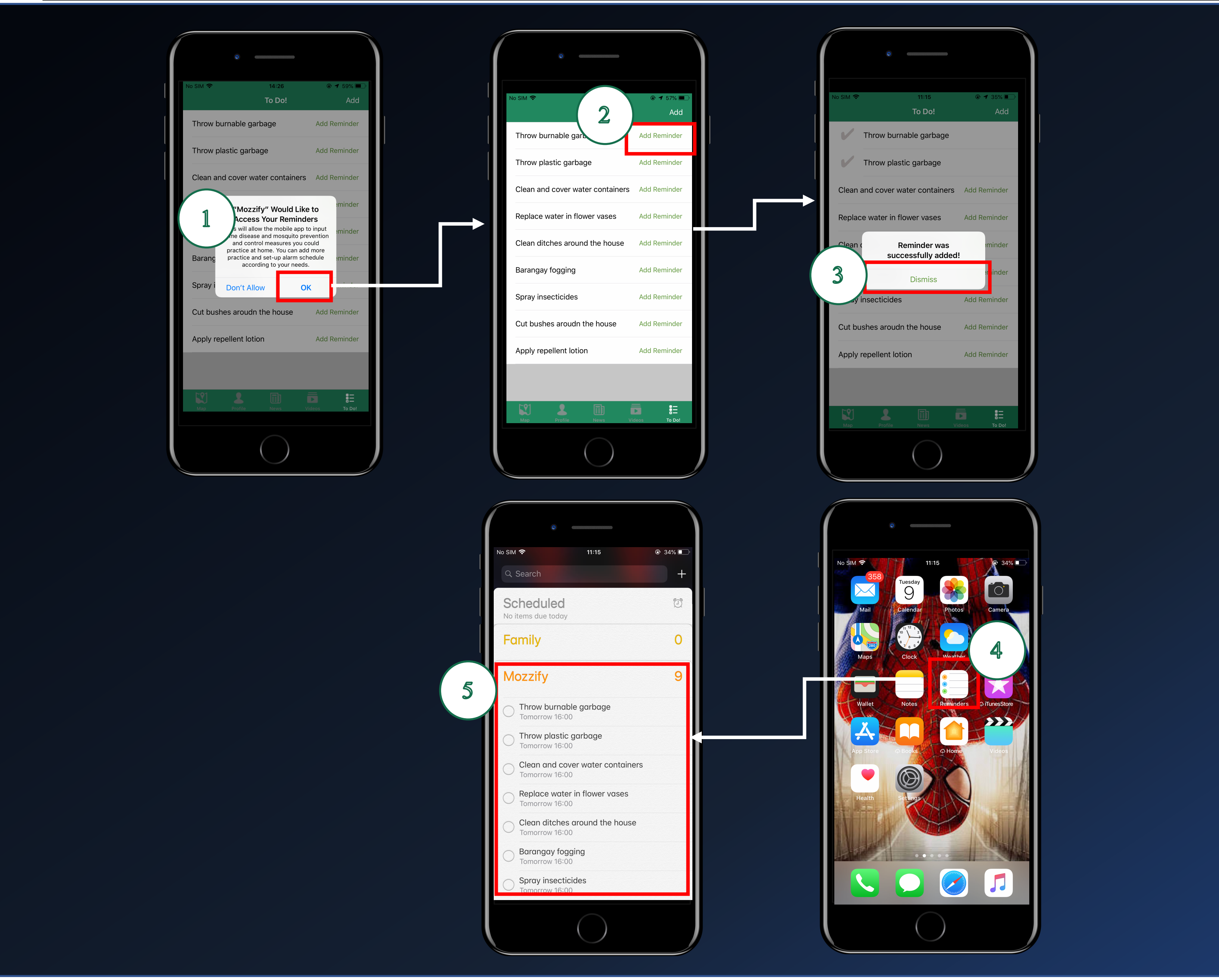

1. Click ***OK*** button to allow **Mozzify** to make a reminder list.

2. Click ***Add Reminder*** button to add each preventive practice to the reminder list.

3. Click ***Dismiss*** button for the alert to disappear.

4. Go to the **Reminders app** in your mobile phone.

5. **Mozzify** reminder list will be added to your reminders.

All rights reserved. No part of this publication may be produced or transmitted in any form or by any means, including photocopying and recording, without seeking the permission of the developer. For inquiries, email herbuelavonralphdane@gmail.com

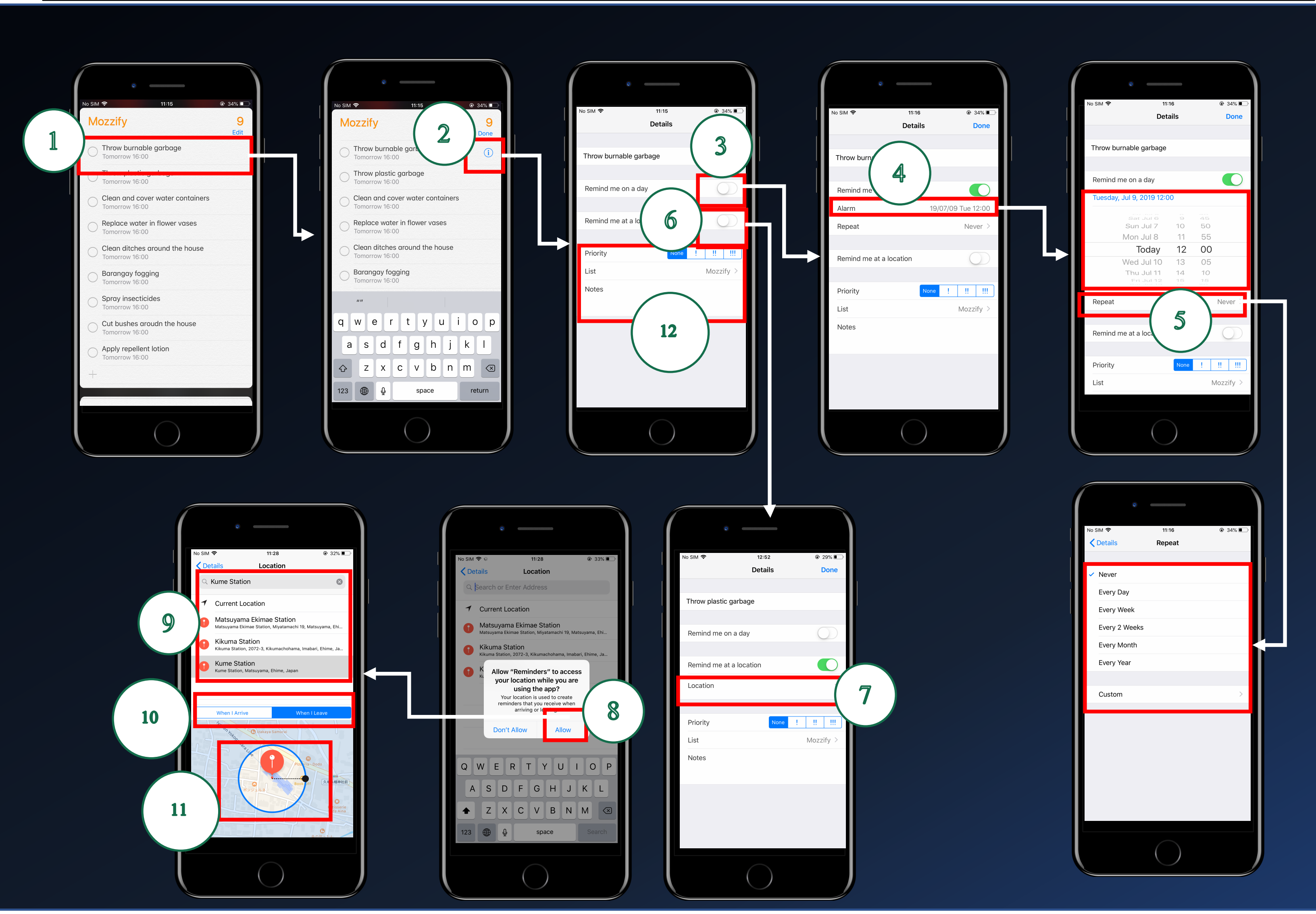

1. Click the **preventive practice** that you want to edit settings.

2. Click to show **information**.

3. Click to set **day**.

4. Click to set **date and time**.

5. Click to edit **Repeat** options.

6. Click to set **location**.

7. Click to go to **location settings**.

8. Click ***Allow*** to proceed to location settings.

9. Choose among the **options** or **enter a new location** or click **current location**.

10. Choose either set the reminder to **When I arrive** or **When I leave**.

11. Set the **vicinity radius** by tapping and holding the **black dot** in the **blue circle** then click ***Details*** button to go back.

12. Set **Priority** and add **Notes**, then click **Done** button to save.

All rights reserved. No part of this publication may be produced or transmitted in any form or by any means, including photocopying and recording, without seeking the permission of the developer. For inquiries, email herbuelavonralphdane@gmail.com

9. To Do

9.3. Adding New Practice, Completing and Deleting a Practice

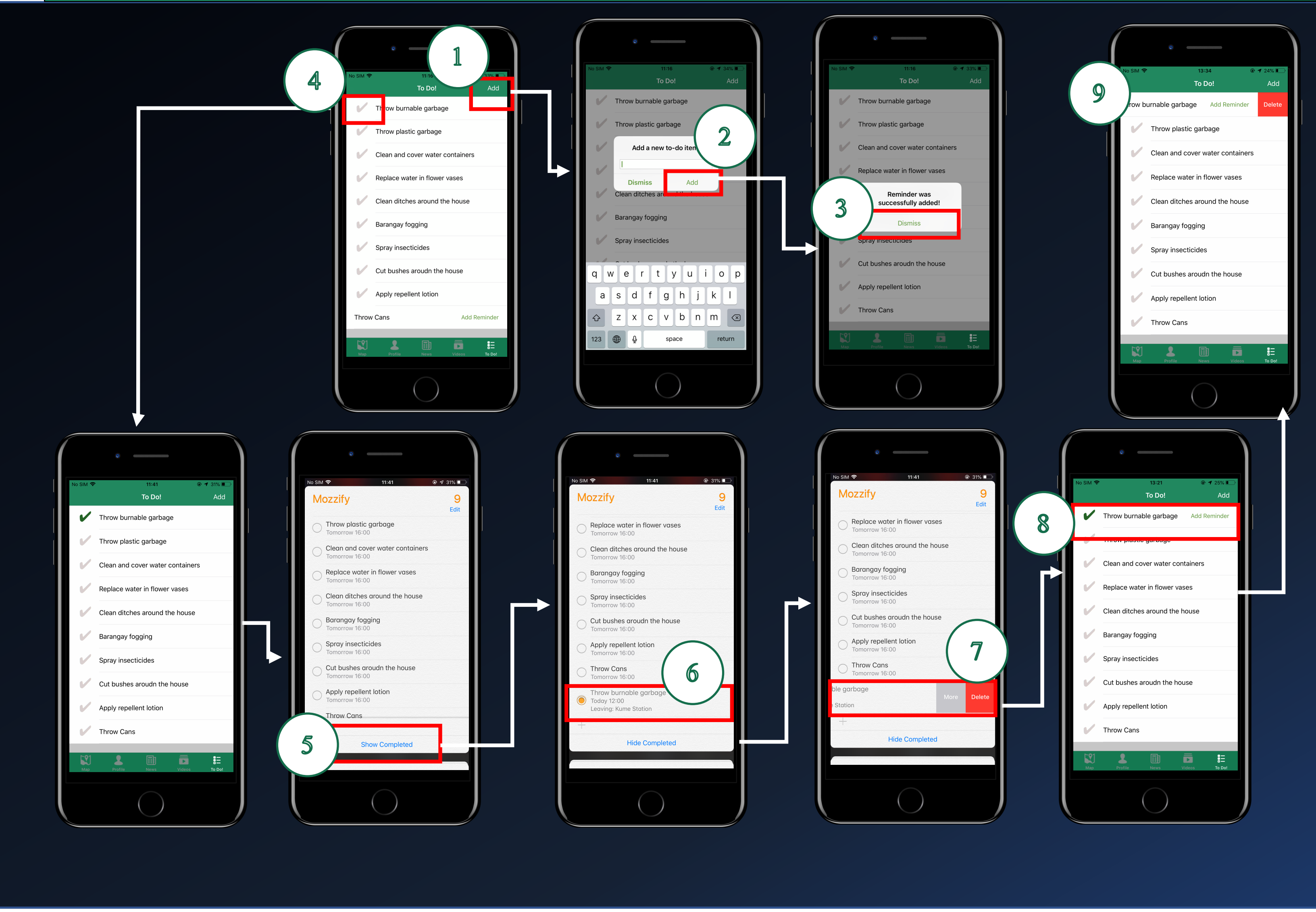

<u>Adding new practice:</u>

1. Click ***Add*** button

2. Enter **new practice** in the text field and click ***Add***.

3. Click ***Dismiss.***

<u>Completing a practice:</u>

4. Click the checkmark button.

5. Go to Reminders app then click Show Completed button.

6. The completed practice is ticked.

<u>Deleting a practice:</u>

In the Reminders app:

7. Swipe the practice to the left then click ***Delete*** button.

In the Mozzify app:

8. Go back to Mozzify app and see the deleted practice has ***Add Reminder*** button again.

9. Swipe the practice left then click ***Delete***.

All rights reserved. No part of this publication may be produced or transmitted in any form or by any means, including photocopying and recording, without seeking the permission of the developer. For inquiries, email herbuelavonralphdane@gmail.com

ArcGIS ESRI Map (Data Collection)
https://github.com/Esri/data-collection-ios

ArcGIS Runtime Toolkit
https://github.com/Esri/arcgis-runtime-toolkit-ios

News (News-App)
https://github.com/johnnyperdomo/News-App

Video (My Videos), File: 30DSC-Youtube-Day2-UITableView
Youtube: https://www.youtube.com/watch?v=RAFsNJ-qjsg

Videos (Youtube)
https://www.youtube.com/watch?v=VC9Kl182E-E&t=13s
https://www.youtube.com/watch?v=pABkc9mX_0M&t=10s
https://www.youtube.com/watch?v=uw-TgokvEi4
https://www.youtube.com/watch?v=cnU-npubpKw
https://www.youtube.com/watch?v=rEyslDif1o4&t=17s
https://www.youtube.com/watch?v=o9y-5VKJRiQ
https://www.youtube.com/watch?v=I2G2o-oJHl0
https://www.youtube.com/watch?v=WUNDs3RLN-U&t=316s
https://www.youtube.com/watch?v=VC9Kl182E-E&t=2s
https://www.youtube.com/watch?v=9KzMjjgFmKc&t=23s

LoginView and Timeline
Part 1https://www.youtube.com/watch?v=UPKCULKi0-A
Part 2 https://www.youtube.com/watch?v=gWZP0vDgMtg
Part 3 https://www.youtube.com/watch?v=n6eR9lJDiyY
Part 4 https://www.youtube.com/watch?v=csz_mFklOek
Part 5 https://www.youtube.com/watch?v=R95eeehl8Ck
Part 6 https://www.youtube.com/watch?v=vgoYNswX6C8
Part 7 https://www.youtube.com/watch?v=-qoCHVwGn18
Part 8 https://www.youtube.com/watch?v=4y_NLkYT6NU
Part 9 https://www.youtube.com/watch?v=nSBWySBDj3E

To Do: Reminder App
https://www.raywenderlich.com/2291-eventkit-tutorial-making-a-calendar-reminder

PDF Reader
https://github.com/taminhtu/PDFReader

Web (Youtube)
https://www.youtube.com/watch?v=C0Z6tJdeQ_E&t=7s

All rights reserved. No part of this publication may be produced or transmitted in any form or by any means, including photocopying and recording, without seeking the permission of the developer. For inquiries, email herbuelavonralphdane@gmail.com

Carousel Effect:
https://www.youtube.com/watch?v=JG7mWFcU0vk

Slider:
https://www.youtube.com/watch?v=yHzaMR5Rwow

App icon:
https://www.youtube.com/watch?v=zHq3zBjCMtY

Map Directions
https://www.youtube.com/watch?v=rmafKbCHKe0

Storing Data (User defaults)
https://www.youtube.com/watch?v=S69sxZELJRQ&t=322s

Segmented control:
https://www.youtube.com/watch?v=S69sxZELJRQ&t=322s

PDF
https://www.who.int/tdr/publications/documents/dengue-diagnosis.pdf

http://www.pgh.gov.ph/static/media/uploads/documents/clinicaldepartments/pediatrics/denguelecture/6clinical.pdf

http://www.pidsphil.org/home/wp-content/uploads/2017/06/2017_Dengue_CPG_Final.pdf

http://apps.searo.who.int/PDS_DOCS/B4751.pdf

https://www.doh.gov.ph/sites/default/files/statistics/Dengue%20Monthly%20Report_MW1-MW30_2018_No.7.pdf

All rights reserved. No part of this publication may be produced or transmitted in any form or by any means, including photocopying and recording, without seeking the permission of the developer. For inquiries, email herbuelavonralphdane@gmail.com
